# Genetic dissection of the gene coexpression network underlying photosynthesis in *Populus*

**DOI:** 10.1111/pbi.13270

**Published:** 2019-10-21

**Authors:** Liang Xiao, Xin Liu, Wenjie Lu, Panfei Chen, Mingyang Quan, Jingna Si, Qingzhang Du, Deqiang Zhang

**Affiliations:** ^1^ Beijing Advanced Innovation Center for Tree Breeding by Molecular Design Beijing Forestry University Beijing China; ^2^ National Engineering Laboratory for Tree Breeding College of Biological Sciences and Technology Beijing Forestry University Beijing China; ^3^ Key Laboratory of Genetics and Breeding in Forest Trees and Ornamental Plants Ministry of Education College of Biological Sciences and Technology Beijing Forestry University Beijing China

**Keywords:** association genetics, coexpression, eQTN, epistasis, *PtoPsbX1*, photosynthesis

## Abstract

Photosynthesis is a key reaction that ultimately generates the carbohydrates needed to form woody tissues in trees. However, the genetic regulatory network of protein‐encoding genes (PEGs) and regulatory noncoding RNAs (ncRNAs), including microRNAs (miRNAs) and long noncoding RNAs (lncRNAs), underlying the photosynthetic pathway is unknown. Here, we integrated data from coexpression analysis, association studies (additive, dominance and epistasis), and expression quantitative trait nucleotide (eQTN) mapping to dissect the causal variants and genetic interaction network underlying photosynthesis in *Populus*. We initially used 30 PEGs, 6 miRNAs and 12 lncRNAs to construct a coexpression network based on the tissue‐specific gene expression profiles of 15 *Populus* samples. Then, we performed association studies using a natural population of 435 unrelated *Populus tomentosa* individuals, and identified 72 significant associations (*P* ≤ 0.001, *q* ≤ 0.05) with diverse additive and dominance patterns underlying photosynthesis‐related traits. Analysis of epistasis and eQTNs revealed that the complex genetic interactions in the coexpression network contribute to phenotypes at various levels. Finally, we demonstrated that heterologously expressing the most highly linked gene (*PtoPsbX1*) in this network significantly improved photosynthesis in *Arabidopsis thaliana*, pointing to the functional role of *PtoPsbX1* in the photosynthetic pathway. This study provides an integrated strategy for dissecting a complex genetic interaction network, which should accelerate marker‐assisted breeding efforts to genetically improve woody plants.

## Introduction

Photosynthesis is a crucial process that determines the rate of carbon dioxide fixation in green plants and ultimately the levels of carbohydrates that are used to produce woody tissues in trees (Schaedle 1975; Niinemets 2014). Improving our understanding of the molecular mechanism of photosynthesis is essential for increasing plant growth and biomass accumulation. Recent studies have shown that the genetic modification of genes involved in the photosynthetic pathway can alter the maximum photosynthetic efficiency and biomass of plants (Heyneke & Fernie 2018). Simultaneously overexpressing a set of genes involved in the photosynthetic pathway in tobacco (*Nicotiana tabacum*), that is overexpression of the *sedoheptulose‐1,7‐bisphosphatase* (*SBPase*), *cyanobacterial putative‐inorganic carbon transporter B* (*ictB*) and *fructose‐1,6‐bisphosphate aldolase* (*FBPase*), increased the assimilation rate and biomass increase to a greater degree than wild type (Simkin *et al.*, 2015).

Several transcriptomic and proteomic studies have analysed the interplay between photosynthesis and biotic and abiotic stress tolerance in perennial trees with the aim of uncovering the regulatory mechanism of stress‐induced suppression of photosynthesis (Bernacki *et al.*, 2018; Chen 2018). The roles of several stress response genes in regulating photosynthesis have been identified via transgenic analysis in perennial trees. For example, silencing the expression of the stress response gene *ENHANCED DISEASE SUSCEPTIBILITY1* (*EDS1*) improved the quantum yield of photosystem II and chlorophyll content in hybrid *Populus* (*Populus tremula* L. × *P. tremuloides*) (Bernacki *et al.*, 2018). Although several studies have demonstrated the important roles of various individual genes in the photosynthetic pathway, the genetic architecture of the targeted photosynthesis‐related traits remains unknown, especially the magnitude of the genetic effects of causal genes and alleles, as well as the allelic interactions among genes involved in the photosynthetic pathway.

With the rapid development of RNA‐sequencing (RNA‐seq) technologies, numerous noncoding RNAs (ncRNAs), especially microRNA (miRNAs) and long noncoding RNAs (lncRNAs), which play critical regulatory roles in plant development, growth processes and stress responses, have been identified in many plant species (Ariel *et al.*, 2015). Emerging evidence indicates that ncRNAs are important genetic regulators of target gene expression (Kim & Sung 2012; Liu *et al.*, 2015). miRNAs are capable of controlling homeostasis and significantly contribute to the degradation of mRNA and post‐translational inhibition through complementary base pairing (Chen 2005). For instance, the overexpression of miR408, a negative regulator of *UCLACYANIN8* (*UCL8*), a member of the photocyanin family, enhanced photosynthesis and enlarged seed size in *Arabidopsis thaliana*, *N. tabacum* and *Oryza sativa* (Pan *et al.*, 2018). Although the regulatory mechanism of plant lncRNAs is unclear, some studies have indicated that lncRNAs regulate gene expression in *cis* or in *trans* and function through transcriptional or post‐transcriptional mechanisms (Ariel *et al.*, 2015; Kim & Sung 2012). For example, HID1, a conserved 236‐nucleotide (nt) lncRNA in land plants, represses the transcription of *At‐PIF3* and function as a key repressor of seedling photomorphogenesis (Wang *et al.*, 2014). These studies suggest that ncRNAs are key regulators with important roles in the photosynthetic pathway.

Coexpression networks comprise gene clusters with highly similar expression profiles that function jointly in various biological contexts and are involved in similar biological pathways or are subjected to shared regulatory pathways (Eisen *et al.*, 1998). Offering a wealth of intuitive concepts for describing the biological interactions among genes, coexpression networks have been constructed based on functionally related genes in several plant species, including *A. thaliana*, *Zea mays* and *Populus tremula* (Obayashi *et al.*, 2008; Schaefer *et al.*, 2014; Sundell *et al.*, 2017). For instance, Sundell *et al. *(2017) identified the conserved gene coexpression modules underlying distinct biological processes involved in wood formation in *Populus* under diverse experimental conditions, genotypes and treatments. To improve our understanding of the genetic regulatory mechanisms of the photosynthetic pathway, it is critical to uncover the interaction networks involving protein‐encoding genes (PEGs) and ncRNAs (lncRNAs and miRNAs) underlying photosynthesis and biomass accumulation in trees.

Association mapping is a genetic approach that has been used to dissect the natural allelic variation responsible for complex quantitative traits in perennial trees (Du *et al.*, 2019). The genetic variation of quantitative traits is classified into additive, dominance and epistatic effects, which are conferred by numerous genes/alleles in multiple biological networks (Du *et al.*, 2015). Additive and dominance association models are widely used to determine the putative functional variants in biological pathways and the genetic architecture of complex traits (Evans *et al.*, 2014). Epistasis is the interactive effects among multiple loci or genes that contribute to the same phenotype (Mackay & Moore 2015). Epistasis association studies have revealed gene—gene interactions underlying complex traits (Hu *et al.*, 2015). For instance, Phan *et al. *(2016) demonstrated that the epistatic interactions among *SnToxA*, *SnTox1* and *SnTox3* play important roles in determining resistance to *Septoria nodorum* blotch disease in *Triticum aestivum*. In addition, we previously dissected the effects of the interactions among PtomiR475b‐*PPRs* (*Pentatricopeptide repeat proteins*), as well as PtomiR167a‐ARFRL‐*ARF8* (*Auxin‐related factor 8*), on plant growth and wood formation in *Populus* (Quan *et al.*, 2018; Xiao *et al.*, 2017). These studies demonstrated the power of association analysis for exploring the putative functions of genes and the interactions among multiple genes in specific pathways underlying phenotypic variation.

Expression quantitative trait nucleotide (eQTN) analysis, a technique used to associate allelic variants with gene expression traits, can help unravel the common casual variants that alter the transcript levels of many genes (Majewski & Pastinen 2011). In addition to providing insights into the biology underlying the roles of genes in regulating phenotypes, eQTN mapping combined with association studies has been used to dissect the genetic architecture of quantitative traits in several species, including maize and tomato (Fu *et al.*, 2013; Gilad *et al.*, 2008; Zhu *et al.*, 2018). The combination of association mapping (additive, dominance and epistasis) and eQTN analysis can be used to uncover genetic interactions among multiple gene regulatory networks of photosynthetic traits.

Here, we used coexpression analysis to identify the putative regulatory network (including PEGs, lncRNAs and miRNAs) involved in the photosynthetic pathway of *Populus*. By integrating association studies (additive, dominance and epistasis) with eQTN mapping, we deciphered the genetic basis of these coexpressed genes and their potential functional relevance to photosynthesis in a natural population of 435 unrelated *Populus tomentosa* individuals. We also characterized the function of a hub gene via overexpression in *A. thaliana*, which further elucidated the genetic regulatory network and casual genes involved in photosynthesis. This information could be used to create strategies to improve photosynthetic efficiency and biomass accumulation in *P. tomentosa.*

## Results

### Annotation of the coexpression module of photosynthetic PEGs in *Populus*

To search for gene transcripts involved in the core photosynthetic pathway, we investigated the overall transcriptional activity in the leaves of 1‐year‐old *P. tomentosa* clone ‘1316’ via RNA‐seq and detected 21 366 expressed PEGs in leaves. We performed Gene Ontology (GO) enrichment analysis and determined that 94 PEGs clustered into three GO terms related to the biological process of photosynthesis (GO: 0009765, GO: 0015979 and GO: 0019684) (Table S1), suggesting that these PEGs are involved in the photosynthetic pathway. To help uncover the putative genetic regulatory network involved in photosynthesis in poplar, we constructed a coexpression module based on gene expression of 94 PEGs of 15 samples from different poplar genotypes (including the 14 *Populus trichocarpa* tissues described by Rodgers‐melnick *et al. *(2012) and one leaf tissue from *P. tomentosa* clone ‘1316’ described above.). Under the threshold of |ρ| > 0.95 and *P* < 0.001, we identified that 30 PEGs constructed a significantly correlated expression module (Table S2). Based on annotation information, we identified that all 30 PEGs share the same regulatory pathway and functional coordination involving in photosynthesis (Table S3).

### Identification of regulatory ncRNAs of the photosynthesis‐related PEGs in *Populus*

**Figure 1:**
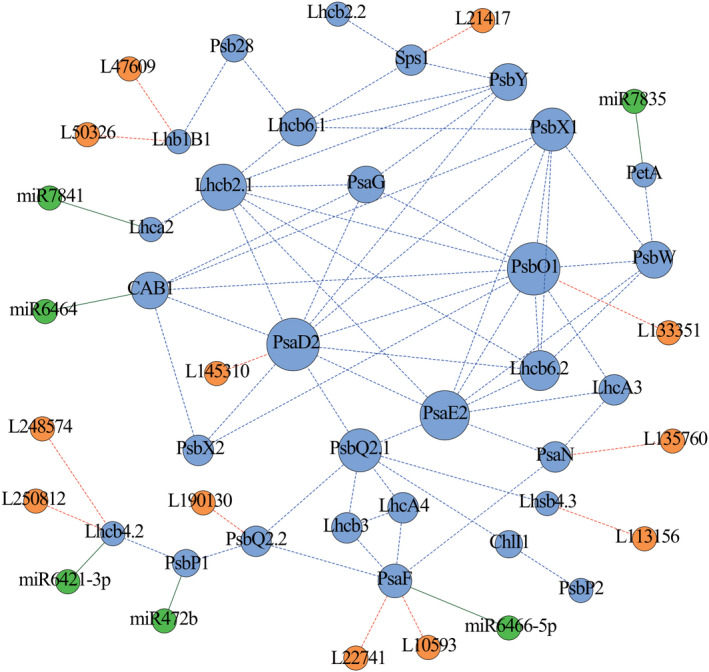
The coexpression network involved in the photosynthetic pathway in *Populus.* The putative regulatory network of PEGs (blue circles), lncRNAs (orange circles) and miRNAs (green circles) constructed by coexpression analysis and ncRNA prediction. The blue, orange and green lines represent the PEG–PEG, lncRNA‐PEG and miRNA‐PEG interactions, respectively. The dotted lines indicate potential interactions, including coexpression interactions and the interactions of lncRNAs with their putative target PEGs; the solid lines indicate interactions verified by degradome sequencing.

We explored ncRNAs involved in regulating the expression of the 30 PEGs in the coexpression module and identified 12 lncRNAs‐mRNA pairs representing 9 photosynthetic PEGs and 12 lncRNAs from clone ‘1316’ of *P. tomentosa* (Table S4). In addition, using the psRNATarget server combined with degradome sequencing, we detected six PEGs that are regulated by six miRNAs (Figure S1A and Table S5). The miRNA‐binding sites in three of the PEGs were located in the 3'‐untranslated regions, whereas the remaining three were located in exons. Finally, 30 PEGs, 12 lncRNAs and 6 miRNAs were used to construct an integrated network (Figure 1), which illustrated the possible regulatory relationship underlying photosynthesis.

### The candidate genes exhibit high nucleotide diversity and rapidly declining linkage disequilibrium (LD)

Genomic resequencing of 435 individuals from the association population of *P. tomentosa* revealed 2, 130, 631 and 97 common SNPs (minor allele frequency (MAF)>5% and max missing <20%) from 30 PEG, 12 lncRNA and 6 miRNA genes, respectively (Table S6). 235 SNPs were annotated to the exon, and 81 SNPs caused non‐synonymous substitutions in PEGs. Finally, 11 SNPs were identified in the precursor regions of five miRNA genes. For example, PtoMIR6466_SNP2 in the mature region of PtomiR6466 altered the secondary structure of this miRNA, leading to the change of stem‐loop structure and increasing the minimum free energy from −81.80 to −77.10 kcal/mol (Figure S1B). We also predicted that the mutation due to the presence of the SNP in the mature region of PtomiR6466 would prevent this miRNA from cleaving its target site, with the expected value given by psRNATarget software increasing from 4.0 to 7.0.

To evaluate the overall patterns of LD for each gene, we pooled estimates of the squared allele frequency correlations (*r* ^2^) for all pairwise SNP combinations within each gene using Tassel 5.0 software. Nonlinear regression analysis indicated that *r^2^* rapidly dropped to 0.1 within ~ 200 to ~ 600 bp, indicating that the LD decay does not extend over the entire gene region (Figure S2). Different genes showed different levels of nucleotide diversity, with Pi (π) values ranging from 0.004 to 0.146 and Theta (θ) values ranging from 0.007 to 0.180, indicating that these 48 genes are subjected to different levels of selection pressure (Table 1 and Table S7).

**Table 1:** Summary of SNPs and nucleotide diversity within the candidate genes

Category	Gene number	SNP number	Frequency (bp‐1)	Nucleotide diversity	
				π	θw
PEGs	30	2100	25–293	0.004–0.124	0.07–0.180
lncRNAs	12	667	32–544	0.028–0.114	0.058–0.143
miRNAs	6	91	49–217	0.012–0.146	0.027–0.149

### Allelic variation significantly affects photosynthetic efficiency in the *P. tomentosa* association population

To further explore the allelic variants for photosynthetic efficiency in the coexpression network, we measured 24 traits from the functional leaves of 435 individuals in the association population of *P. tomentosa*. Based on the characteristic of traits, they were broadly classified into five categories: photosynthetic characteristics, leaf shape, leaf biomass, pigment content and enzyme activities. The coefficient of variation (CV) was used to assess the variability of the traits. All 24 traits exhibited high phenotypic diversity, with CV values ranging from 0.12 (leaf width [LW], 7.86 ± 0.93 cm) to 3.669 (stomatal conductance [Cond], 0.22 ± 0.81 mol H2O/m^2^/s) (Table S8). However, we detected distinct differences in CV among the five categories, with the lowest phenotypic diversity for leaf size traits and the highest for enzyme activity traits (Table S8). We next assessed correlations among the 24 phenotypic trait values using Pearson’s correlation analysis. Traits within the same category were often closely correlated, except for some traits in the enzyme activity category (Figure S3 and Table S9). These results indicated that the 435 unrelated individuals possessed significant genetic variability and could be used for population genetic analyses.

**Figure 2:**
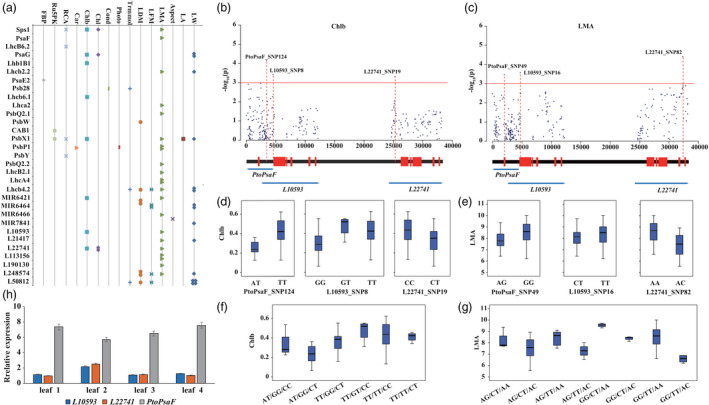
Allelic loci within photosynthetic genes across the coexpression network significantly affecting photosynthetic efficiency. (a) Scatterplot of significant SNP‐trait associations via single SNP‐based associations (*P* < 0.001). The points represent traits, and multiple points indicate that several SNPs are associated with the same gene. The x‐axis represents traits, and the y‐axis represents genes. (b) Manhattan plot for Chlb content showing *PtoPsaF* and its regulatory lncRNAs (*L10593* and *L22741*). Genes are shown at the bottom (red rectangles, transcribed sequences; black lines, nontranscribed sequences). (c) Genotype effects of each causal SNP from *PtoPsaF* and its regulatory lncRNAs for Chlb content. (d) Six possible genotype combinations with a frequency of ≥5% from the three allelic variations, and the effects of genotype combinations on Chlb content in the association population of *P. tomentosa*. The SNPs in each genotype combination are shown in order according to (c). (e) Manhattan plot for LMA showing *PtoPsaF* and its regulatory lncRNAs (*L10593* and *L22741*). (f) Genotype effects of each causal SNP from *PtoPsaF* and its regulatory lncRNAs for LMA. (g) Eight possible genotype combinations with a frequency of ≥5% from the three allelic variations and the effects of genotype combinations on LMA. The SNPs in each genotype combination are shown in order according to (f). (h) The expression patterns of *L10593*, *L22741*, and *PtoPsaF* in four tissues of *P. tomentosa.*

We then employed a mixed linear model (MLM) association mapping approach to examine the additive/dominant effects between 2,858 common SNPs and 24 photosynthesis‐related traits. We identified 59 unique SNPs in 31 genes (20 PEGs, seven lncRNAs and four miRNAs) that were significantly associated with 16 traits (*P* < 0.001) (Figure 2a, Figure S4–S6). Each SNP explained phenotypic variance (*R* ^2^) ranging from 0.91% to 24.81% (average *R* ^2^ = 10.50%) (Table S10). Moreover, only seven significant SNPs were located in the coding regions of the PEGs, indicating that the allelic variants in noncoding region of the genes may relatively predominate affecting the phenotypic variation.

Among the significant SNP–trait associations, 44 out of 72 associated loci showed significant additive effects, 43 showed dominant effects, and 16 showed both additive and dominant effects (Table S10). Furthermore, eight SNPs were simultaneously associated with two or three traits. These results indicate that these genes have pleiotropic effects. For instance, PtoPsbX1_SNP34, located in the 5′‐untranslated region of *PtoPsbX1*, is simultaneously associated with LW (additive and dominant, *R* ^2^ = 24.81%), and ribulose‐5‐P‐kinase (Ru5PK; dominant, *R* ^2^ = 14.65%), indicating that this SNP has additive or dominant effects on different traits and that *PtoPsbX1* has pleiotropic effects.

We also detected multiple genes associated with the same trait and determined that these genes are regulated by their associated ncRNAs and mRNAs. *PtoPsaF* is the *cis* target of *L10593* and *L22741*. *PtoPsaF* and both lncRNAs genes are associated with chlorophyll b content (Chlb) (Figure 2b) and leaf dry mass of per area (LMA) (Figure 2c). The significant SNPs had different genetic effects (Figure 2d,e), and different allele/genotype combinations had different effects on each trait (Figure 2f,g). We also detected a strong negative correlation between the expression level of *PtoPsaF* versus *L10593* and *L22741* (Figure 2h). These findings indicate that *PtoPsaF* and its corresponding lncRNAs are involved in the same pathway to modulate photosynthesis. Finally, the SNPs PtoMIR6466_SNP2 and PtoPsaF_SNP49 were both associated with LMA (*P* = 6.37 × 10^−4^ and 3.42 × 10^−4^, and *R* ^2^ = 21.06% and 4.41%, respectively), within additive effects. As mentioned above, PtoMIR6466_SNP2 is a deleterious locus that alters the secondary structure of this miRNA and prevents it from targeting *PtoPsaF* (Figure S1B), indicating that the mutation in this miRNA transcript sequence plays an important role in the cleavage process. These results indicate that ncRNAs are key regulators of photosynthetic efficiency.

### Epistasis revealed the genetic interaction network of photosynthesis

**Figure 3:**
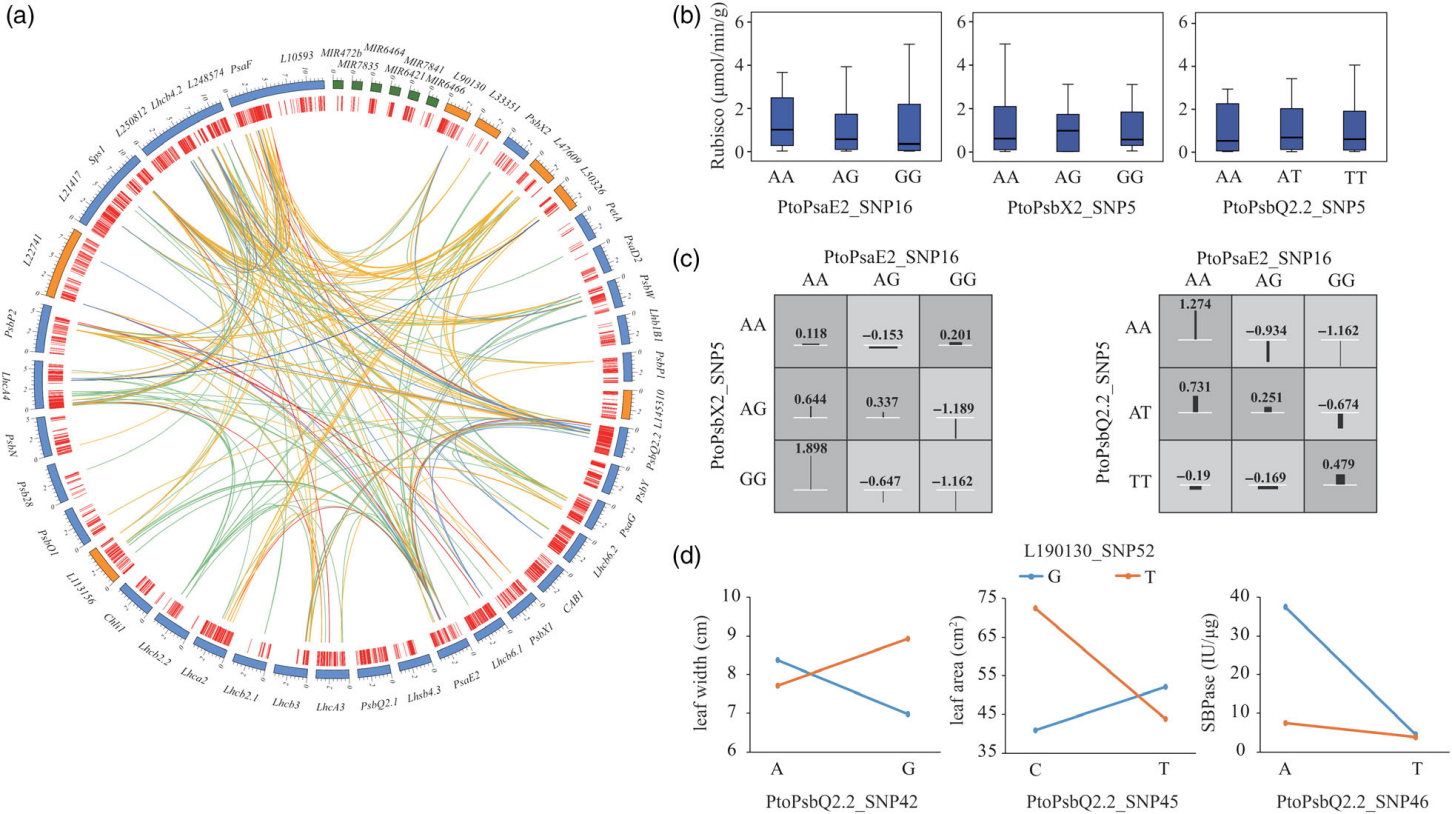
Allelic interactions between significant pairwise SNPs within candidate genes related to the photosynthetic coexpression network. (a) Circos plot showing 511 pairwise interactions for photosynthesis‐related traits (*P* ≤ 0.001). The outer circle represents the PEGs (blue), lncRNAs (orange) and miRNAs (green). The middle circle indicates the positions of the SNPs (red). The interior lines represent the pairwise interactions underlying five categories of traits; the coloured lines represent different categories (orange, red, dark blue, light blue and light green indicate leaf size, leaf mass, photosynthetic characters, pigment content and enzyme activity, respectively). (b) Box plots showing the single SNP genotype effects of PtoPasE2_SNP16 with two loci in *PtoPsbX2* and *PtoPsbQ2.2* for Rubisco activity, and (c) The epistatic effects of different genotypic combinations for Rubisco activity. (d) The epistatic effects for leaf width between L190130_SNP52 with three loci in *PtoPsbQ2.2.*

We identified 511 significant epistatic SNP–SNP pairs for 24 photosynthetic traits (*P* < 0.001), including 185 unique SNPs from 32 genes (23 PEGs and 9 lncRNA genes) (Figure 3a and Table S11). Among these 185 unique SNPs, 133 showed epistatic interactions with more than one SNP. For example, PtoPsaE2_SNP16, located in the promoter region, had 25 epistatic interactions with 24 SNPs underlying enzyme activities, which was not identified by the single SNP‐based association model, indicating that these SNP–SNP pairs have more substantial epistatic effects than single SNPs with additive and/or dominant effects for a particular trait.

Based on the epistasis of SNP–SNP pairs, we constructed the epistasis network among PEGs and ncRNAs (Figure 3a). For example, *PtoPsaE2* is a hub gene linked with 15 PEGs and five lncRNAs that underlie different traits (Table S11). Moreover, different genotypic combinations of SNP–SNP pairs have different epistatic effects, improving our understanding of the epistatic network underlying photosynthesis. For example, *PsaE2* (PtoPsaE2_SNP16) has epistatic interactions with *PtoPsbX2* (PtoPsbX2_SNP5) and *PtoPsbQ2.2* (PtoPsbQ2.2_SNP3), which are associated with Rubisco (Figure 3b); the phenotypic values of various genotypic combinations were different from those of single SNP effects (Figure 3c). Interestingly, the epistatic interactions of ncRNAs with their targets can affect multiple traits. For instance, L190130_SNP52 had epistatic interactions with the three SNPs in *PtoPsbQ2.2*, and these allelic combinations showed nonadditive effects for several traits, which also supports the finding that *L190130* and *PtoPsbQ2.2* share a regulatory relationship (Figure 3d). These results demonstrate that the analysis of epistatic interactions enriches our understanding of the interactions between PEGs and ncRNAs, representing a complementary strategy for dissecting the genetic architecture and identifying the interaction networks underlying photosynthesis in *P. tomentosa*.

### Analysis of the genetic regulatory networks of eQTNs to explore coexpression networks and phenotypic variation

To explore the regulatory interactions between allelic variants and PEG expression, we mapped the 2,858 common SNPs to the transcript abundance of 23 PEGs (expressed in ≥80% of the 435 individuals). At* P* ≤ 0.001 and *q* ≤ 0.05, we detected 174 unique SNPs associated with the gene expression level (Table S12), within 29.9% of the SNPs associated with more than one expressed gene. For example, PtoSps1_SNP79 (*PtoSps1*, encoding solanesyl diphosphate synthase 1) is associated with the transcript levels of six PEGs, suggesting it might be an eQTN hot spot with a strong regulatory effect on the expression of several linked loci, thereby affecting the expression levels of different genes. Notably, only 10.9% of the eQTNs were found in coding regions, suggesting that noncoding sequence variants are major regulators of gene expression. This eQTN analysis identified 100 gene–PEG expression regulatory pairs (Figure S7), indicating that this is a powerful approach for dissecting the interactions among genes in coexpression networks. Among these pairs, 37 represent ncRNAs that regulate the expression of PEGs, indicating that ncRNAs play important roles in the variation in photosynthesis‐related gene expression.

**Figure 4:**
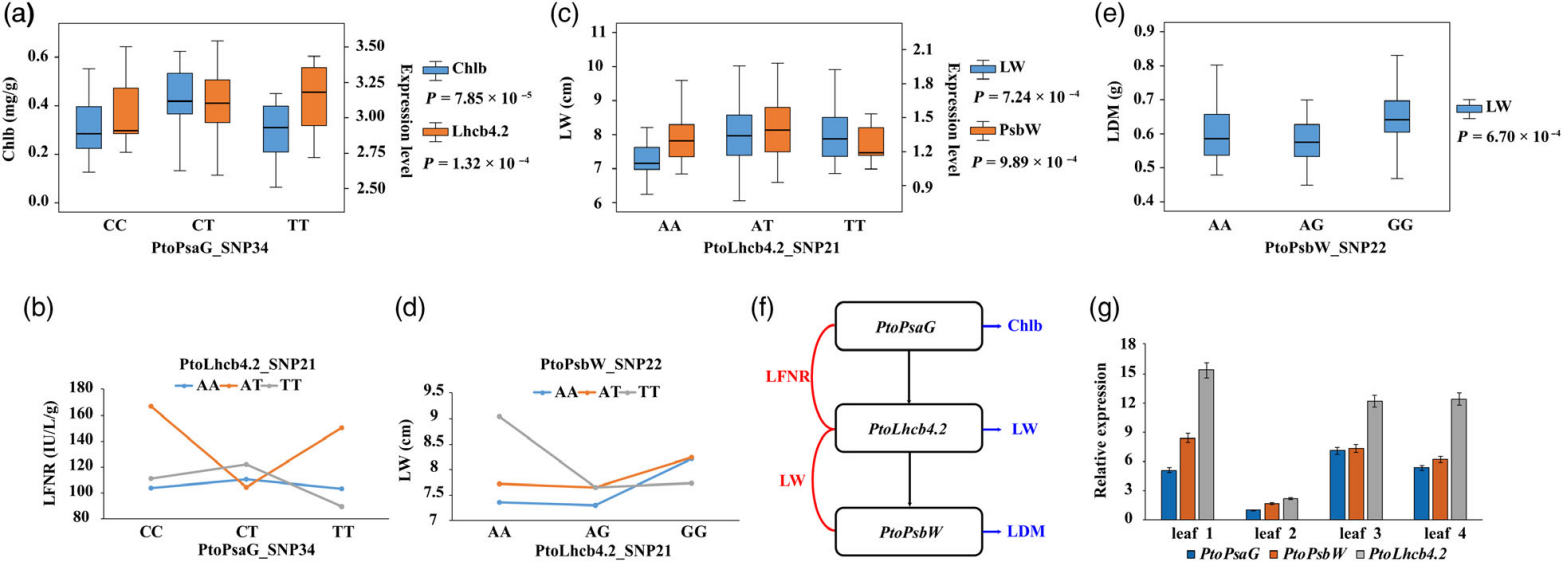
Integration of epistasis and eQTN analysis identifies the important components involved in the photosynthetic pathway. (a) Box plot for the Chlb trait (blue) and *PtoLhcb4.2* expression (orange) plotted as an effect of genotype at *PtoPsaG*_SNP34. (b) Pairwise interactions between PtoPsaG_SNP34 and PtoLhcb4.2_SNP21 control the LFNR trait with different genotypic combinations at the two loci. (c) Box plot for the LW trait (blue) and *PtoPsbW* expression (orange) plotted as an effect of genotype at PtoLhcb4.2*_*SNP21. (d) Pairwise interactions between *PtoLhcb4.2_*SNP21 and *PtoPsbW_*SNP22 control the LFNR trait with different genotypic combinations at the two loci. (e) Box plot for the LDM trait (blue) plotted as an effect of genotype at PtoPsbW_SNP22. (f) The genetic regulatory network of *PsaG‐Lhcb4.2‐PsbW* constructed based on the epistasis and eQTN mapping data. The horizontal blue arrows represent the genes associated with photosynthetic traits via a single SNP‐based association; the vertical black arrows represent the regulatory relationships of eQTNs; the red curves indicate the epistatic interactions of genes underlying complex traits. (g) The expression patterns of *PtoPsaG*, *PtoLhcb4.2* and *PtoPsbW* in four tissues and organs of *P. tomentosa.*

In addition, 33 SNPs detected by single SNP‐based association studies and 65 SNPs detected by the epistasis model overlapped with SNPs identified by eQTN analysis. For example, PtoPsaG_SNP732 was associated with Chlb and *PtoLhcb4.2* expression (Figure 4a), and PtoLhcb4.2_SNP21 might interact with PtoLhcb4.2_SNP34 to control LFNR (Figure 4b), suggesting that *PtoPsaG* affects the expression of *PtoLhcb4.2* to form an epistatic interaction, which would contribute to the phenotypic variation of LFNR. Moreover, the traits associated with PtoPsaG_SNP34 revealed by single SNP‐based association and epistasis analysis were Chlb and LFNR, respectively, revealing that epistasis effects are indispensable components in the genetic architecture of multiple complex quantitative traits. The same relationship was detected between PtoPsaG_SNP21 and PtoPsbW_SNP22 (Figure 4c,d,e). Hence, cascade regulation might be a significant component of the coexpression network (Figure 4f). We examined the expression patterns of these three genes in different tissues and found that they were consistent, supporting the importance of cascade regulation (Figure 4g). Therefore, combining single SNP‐based association analysis, epistasis analysis and eQTN mapping helped us unravel the genetic interactions and genetic basis of the photosynthesis‐related gene coexpression network and its effects on photosynthetic traits in poplar.

### Photosystem II subunit X (*PsbX1*, *Potri.006G144000*) is a hub gene that serves as an important genetic regulator of photosynthetic in *P. tomentosa*

**Figure 5:**
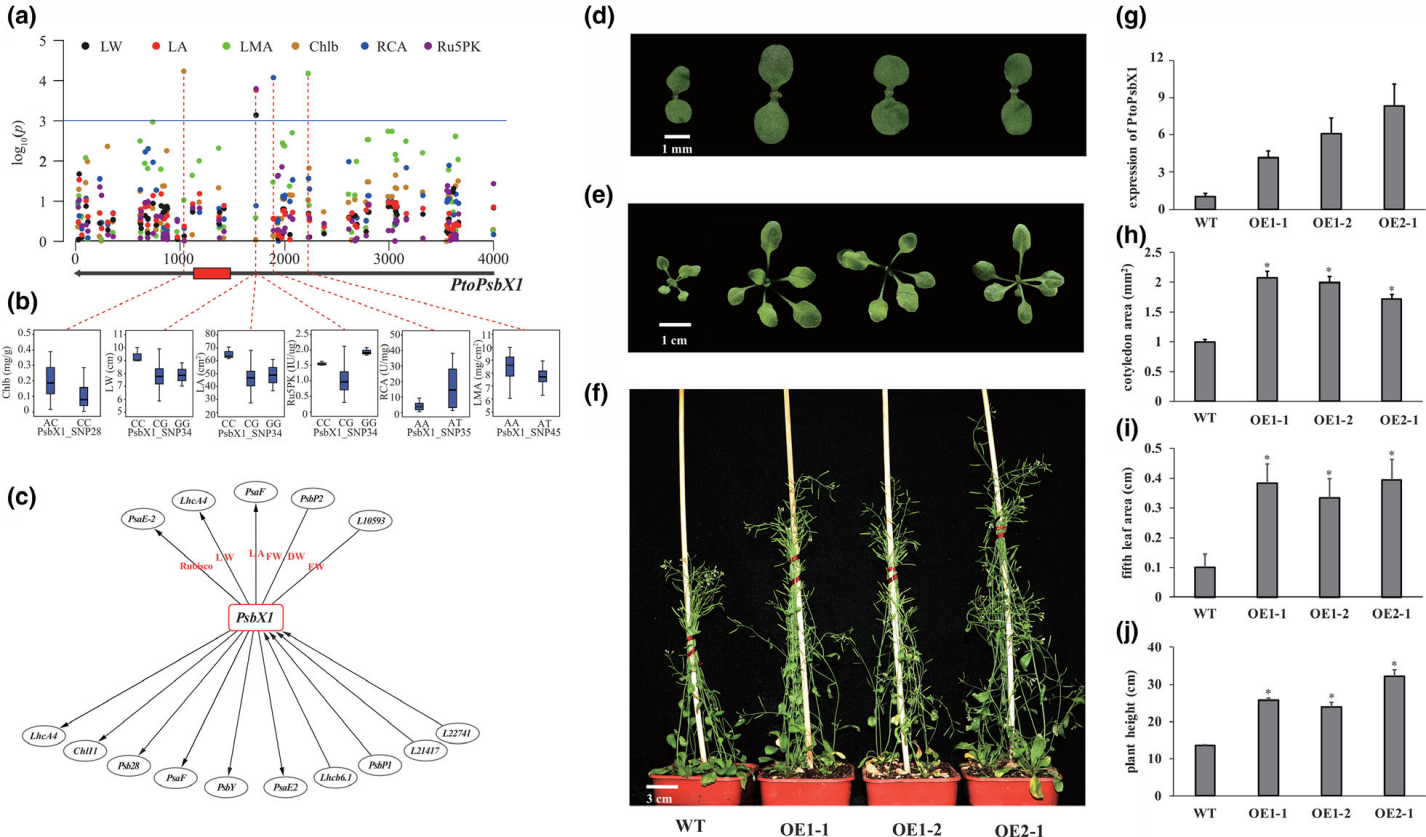
*PtoPsbX1* is a hub gene that functions as an important genetic regulatory factor underlying the photosynthetic pathway in *P. tomentosa.* (a) Manhattan plot displaying the association results between all SNPs of *PtoPsbX1* and six photosynthetic traits. The gene structure is shown below the plot (red rectangle, coding sequences; black lines, noncoding sequences). (b) Box plots showing the genotype effects of each significant SNP of *PtoPsbX1* and its associated traits. (c) *PtoPsbX1* functions as a ‘connector’ linking nine PEGs and three lncRNAs by epistasis and eQTNs in the photosynthetic pathway. The ellipses at the top represent genes that interact with *PtoPsbX1* with epistatic effects underlying photosynthetic traits. The ellipses at the bottom represent gene interactions within eQTNs. The arrows pointing to *PtoPsbX1* indicate genes that regulate the expression of *PtoPsbX1*; the arrows pointing to other genes indicate that *PtoPsbX1* regulates the expression of the PEGs. (d‐f) Morphological comparison of OE‐PtoPsbX1 lines and the wild type (WT). (g) Relative expression levels of *PtoPsbX1* and plant height in four OE‐PtoPsbX1 lines and the WT. (H–J) Quantitative measurement of cotyledon area (h), area of the fifth rosette leaf (i) and mature plant height (j) in the *A. thaliana* lines. *Significant differences from the WT based on Student’s *t*‐test (*P* < 0.01); *n* = 15.

Based on to the classification of 24 phenotypic traits, we analysed significant associations between genes and five classes of phenotypes and explored the involvement of photosynthetic genes in phenotypic plasticity. Half of the genes were associated with two or more classes of phenotypes. *PtoPsbX1* was associated with four phenotype classes (enzyme activity, pigment content, leaf size and leaf biomass) (Figure 5a,b). The orthologous gene of *PtoPsbX1* in *A. thaliana* is *At2g06520*, which encodes photosystem II subunit X (*PsbX*). Coexpression analysis revealed that *PtoPsbX1* is linked to eight genes (Figure 1), indicating that multiple genetic factors function in this photosynthetic system and that *PtoPsbX1* is a key node in the regulatory network of photosynthesis.

We explored the interaction network of *PtoPsbX1* via epistasis analysis and eQTN mapping. Under the epistasis model, we identified six SNP–SNP epistasis pairs, representing four PEGs, and one lncRNA that interacts with *PtoPsbX1* (Figure 5c). eQTN mapping indicated that *PtoPsbX1* regulates the transcription of six PEGs and that *PtoPsbX1* is regulated by two PEGs and two lncRNAs. The identity of three of four genes identified by epistasis analysis was supported by the results of eQTN mapping, suggesting that epistasis has an impact on gene transcript levels. Together, these findings suggest that *PtoPsbX1* is an important genetic regulator of the photosynthetic network.

Finally, we investigated the role of *PtoPsbX1* in regulating photosynthetic efficiency and biomass accumulation. We heterologously overexpressed *PtoPsbX1* in *A. thaliana* and measured photosynthetic efficiency and plant biomass traits in three independent homozygous transformed lines. Compared to wild‐type plants, the morphology of overexpression plants was markedly enlarged (Figure 5d–f), and *PtoPsbX1* was significantly up‐regulated (Figure 5g). Also, we detected that the pigment content was not significantly increased, but the enzyme activities were significantly improved in all three overexpression lines (Figure S8). The cotyledon area, area of the fifth rosette leaf and plant height were increased 195%, 366% and 200% than the wild type, respectively (Figure 5h–j), pointing to the functional importance of *PtoPsbX1* in the photosynthetic pathway.

## Discussion

Photosynthesis is the most important chemical reaction for life on Earth. However, until recently, no suitable methods were available for systematically revealing the genetic regulatory mechanism of the photosynthetic pathway. Reverse genetics studies have revealed some genes that directly regulate photosynthesis, but the overall regulatory network has remained unclear. Here, we combined coexpression analysis, association studies and eQTN mapping to dissect the regulatory network of the photosynthetic pathway in poplar. We identified a key node gene (*PtoPsbX1*) in this network and functionally validated its importance in the photosynthetic pathway. Thus, coexpression analysis combined with genome‐wide association studies is a powerful approach for identifying significant casual genes in a complex pathway. Even though our analysis was based on limited data, our strategy was successfully used to systematically explore the genetic regulatory mechanism of the photosynthetic pathway, providing a theoretical basis for molecular‐assisted breeding of forest trees.

### Allelic variations in photosynthesis‐related genes across the coexpression network substantially affect photosynthetic efficiency

The bioengineering of photosynthesis in plants and the exploitation of currently available sources of genetic variation for photosynthetic traits represent powerful approaches for improving the genetic yield potential of plants (Long *et al.*, 2015; Pan *et al.*, 2018). Thus, an in‐depth understanding of photosynthesis would enable informed, guided improvement of this crucial process via *in silico*‐assisted genetic engineering (Long *et al.*, 2015). Forest trees provide abundant sources of oxygen and have important economic benefits. Nevertheless, the genetic regulatory mechanism of the photosynthetic pathway in perennial trees and its coordinated multigene network remain unclear. It would thus be of great interest to further explore the regulatory mechanism of photosynthesis in trees.

Coexpression analysis is a powerful tool that can be used to identify the modules of transcriptionally coregulated and functionally related genes. For example, Sibout *et al. *(2017) constructed a coexpression network based on the expression data for *Brachypodium* at 43 different developmental stages in eight organs and identified an *F5H* ortholog involved in lignin biosynthesis. In this study, we constructed a coexpression network including 30 PEGs with similar expression patterns that may share the same regulatory pathway involved in photosynthesis in poplar. Moreover, we now have in‐depth knowledge of the ncRNAs (including miRNAs and lncRNAs) that affect photosynthesis and modulate the expression of their target genes at multiple levels using distinct mechanisms (Chekanova 2015). Abundantly expressed miRNAs and lncRNAs whose expression is modulated during stress responses and in specific tissues were previously identified in *Populus*, suggesting that they play key roles in complex biological pathways (Chen *et al.*, 2015; Lu *et al.*, 2005; Song *et al.*, 2013; Tian *et al.*, 2016). In this study, by combining *in silico* prediction, expression pattern analysis and degradome sequencing, we identified 12 lncRNAs and 6 miRNAs associated with the photosynthetic coexpression network of *P. tomentosa* (Figure 1 and Tables S4 and S5), pointing to the regulatory roles of ncRNAs in the photosynthetic pathway. Expression analysis verified the associated expression of the ncRNAs with their putative targets, for example, the strong negative correlation between *PtoPsaF* with *L10593* and *L22741* (Figure 2h). These findings indicate that ncRNAs function in photosynthesis by modulating the transcript levels of photosynthetic PEGs, advancing our understanding of the genetic regulatory network of the photosynthetic pathway.

Due to the rapidly declining LD and abundant phenotypic variations in association populations of *P. tomentosa*, association mapping is a powerful tool for identifying casual genes or allelic variants underlying phenotypic variation (Savolainen & Pyhäjärvi 2007). Here, we detected 59 unique SNPs that were significantly associated with at least one trait by the association studies (Figure 2a). The 23 out of 59 SNPs were detected in lncRNA and miRNA genes, highlighting the importance of ncRNAs in the photosynthetic pathway. The coordinated roles of ncRNAs and PEGs in photosynthesis strongly depend on the diverse genetic effects of the loci. For instance, SNP loci from two lncRNAs (*L10593* and *L22741*) and their target gene (*PtoPsaF*) were associated with Chlb and LMA with different genetic effects, and these SNPs conferred fixed genotype combinations for Chlb and LMA (Figure 2b–g), respectively, suggesting that *PtoPsaF* and these lncRNAs affect photosynthesis by coregulating Chlb and LMA traits.

The finding that the epistatic interactions of *L190130*_SNP52 with *PtoPsbQ2.2* affect multiple traits (Figure 3d) also verifies the important roles of ncRNAs in regulating photosynthesis‐related traits. *AtUCL8*‐miR408 and *AtPIF3*‐HID1 (HID1 is a conserved 236‐nt lncRNA in land plants) are important components of the genetic regulatory network underlying photosynthesis in *A. thaliana* (Pan *et al.*, 2018; Wang *et al.*, 2014), which also supports the regulatory roles of PEGs‐ncRNA in the photosynthetic pathway. These findings advance our understanding of the coordinated networks of miRNAs, lncRNAs and PEGs in the photosynthetic pathway in *Populus*. Our results provide a rich source of candidate SNPs and associated genes for the genetic analysis and breeding of *Populus* for increased photosynthetic efficiency.

### Combined epistatic interaction analysis and eQTN mapping is a powerful approach for dissecting the genetic regulatory mechanisms of the complex network underlying photosynthesis

Recently, genetic networks have been widely used to model complex relationships among a large number of entities. Constructing genetic networks is an excellent method for illustrating the genetic epistasis among numerous gene–gene interactions (Phillips 2008), and the regulatory relationships between allelic variation and expressed genes employed by eQTN mapping. The analysis of epistasis (the significant interactions between genotypes at multiple loci) is an important method for identifying functional allele pairs that contribute to complex traits that is more powerful than single SNP‐based association analysis for population genetics studies (Mackay & Moore 2015; Wan *et al.*, 2013; Wei *et al.*, 2014). Here, we constructed an epistasis network based on the significant epistatic SNP–SNP pairs, which represent interactions among the PEGs and ncRNAs (Figure 3a). For example, allelic combinations of *L190130* and *PtoPsbQ2.2* showed nonadditive effects for several traits (Figure 3d), indicating that epistasis can be explained by interactions between ncRNAs and their targets. It would be useful to dissect the genetic basis of complex traits based on our epistasis network to determine whether the casual genes and the interaction effects of genes could be associated more precisely with the photosynthetic pathway (Shao *et al.*, 2008). Moreover, since the epistatic effects depend on the allele frequencies of the interacting SNPs, rare variants (minor allele frequencies <0.05) should be considered in future studies, which would improve the power to explore the interactions of SNP pairs in natural populations (Mackay 2014).

eQTN mapping is a powerful tool for resolving gene regulatory networks to identify candidate genetic factors that contribute to complex traits (Cubillos *et al.*, 2012). For instance, eQTN mapping has been used to reveal the complex genetic architecture of the regulation of gene expression and the underlying gene regulatory networks and to identify a hot spot related to plant defence and photosynthesis in tomato (Ranjan *et al.*, 2016). In this study, we identified 282 eQTNs associated with the expression of 18 genes, and these eQTNs represent 100 gene–gene pairs, which highlights the complexity of the regulatory network (Figure S7). *PtoPsbY* expression was significantly associated with 17 genetic factors; similarly, *PtoLhcb4.2* regulates the expression of eight genes, providing an illustration of the power of eQTN mapping for identifying active regulatory factors in the complex photosynthetic pathway (Wen *et al.*, 2018). The direct integration of eQTNs with quantitative trait analyses has facilitated the functional interpretation of complex trait association signals in previous studies (Nica *et al.*, 2010). Combined quantitative trait loci (QTL) and expression‐QTL (eQTL) mapping analysis was previously used to construct a genetic regulatory network affecting melon (*Cucumis melo*) fruit quality and to identify a candidate gene (*CmPPR1*) controlling flesh colour intensity, supporting the notion that eQTN analysis is a powerful approach for identifying trait‐related regulatory factors (Galpaz *et al.*, 2018).

Although the construction of gene regulatory networks has indicated that genes can interact at several different levels, the complex network of regulatory factors underlying quantitative traits has remained unclear (Cubillos *et al.*, 2012). Here, we combined coexpression analysis, epistasis association analysis and eQTN mapping to construct the complex regulatory network underlying the photosynthetic pathway in poplar, which revealed the genetic interactions of PEGs and ncRNAs. For example, the identification of a subnetwork of *PsaG‐Lhcb4.2‐PsbW* (Figure 4f), which was overlaid on the both networks, clarified the detailed genetic association between these genes while pointing to the possible functions of these genes in photosynthesis. Although these genes are associated with different phenotypes (LW, LDM, Chlb and LFNR), these traits reflect different aspects of photosynthetic efficiency, shedding light on the genetic interactions between these genes. Therefore, combining coexpression analysis, epistasis association analysis and eQTN mapping is a feasible approach for dissecting the complex regulatory network of quantitative traits, but the underlying genetic interaction mechanism remains to be explored.

### Photosystem genes are important for improving photosynthetic efficiency and biomass accumulation

A recent multitrait analysis performed at different phenotypic levels revealed the pleiotropy and genetic regulation of genetic factors underlying complex traits (Alonso‐Blanco & Méndez‐Vigo 2014). To deepen our understanding of the genetic regulation of photosynthesis, we developed a systematic strategy integrating association mapping (additive, dominance and epistasis) and eQTN mapping to dissect the genetic basis of 24 photosynthesis‐related traits. By mining the integrated network, we determined that the signature SNP of *PtoPsbX1* (Figure 5a,b) is associated with four categories of phenotypes and interacts with 12 genetic factors, as determined by epistasis analysis and eQTN mapping (Figure 5c), pointing to the master regulatory role of *PtoPsbX1* in the photosynthetic pathway. Previous protein structural analysis indicated that PsbX is a subunit of the photosystem II complex with a low molecular mass; however, our results indicate that *PtoPsbX1* is a key node for interactions with PEGs and ncRNAs, suggesting that it may be more beneficial to identify ‘connections’ than ‘components’ of the photosynthetic pathway.

To confirm that *PtoPsbX1* is a casual gene in the photosynthetic pathway, we heterologously expressed this gene in *A. thaliana*. All three transgenic lines showed higher photosynthetic efficiency and biomass accumulation than the wild type (Figure 5h–j and Figure S8), supporting the notion that *PtoPsbX1* is an important regulator of the photosynthetic pathway in *Populus*. In transgenic *Arabidopsis* transformed with a *PsbX* antisense construct, the amount of functional photosystem II was reduced by 30 to 40% (García‐Cerdán *et al.*, 2009), suggesting that *PtoPsbX1* would increase the accumulation of functional photosystem II complex and improve photosynthetic efficiency and biomass accumulation. Climate association studies revealed that *PsbX* is associated with aridity (an important index affecting photosynthesis) in *Pinus taeda* L. (Eckert *et al.*, 2010), which also supports the relative importance of *PtoPsbX1* in the photosynthetic pathway. These findings further confirm the success of our integrated strategy for identifying a significant genetic factor in a complex pathway.

In summary, we explored the regulatory relationships of genes involved in photosynthesis and verified the function of a key *Populus* gene involved in this process via heterologous expression in *A. thaliana*, providing a basis for understanding the complex genetic regulatory process of photosynthesis. However, the mechanisms underlying the interaction between statistical epistasis and eQTNs remain unclear, especially in *Populus*, and detailed functional analyses are needed to verify the biological details underlying these complex pathways (Ingvarsson & Street 2011). With the continued use of genome‐editing techniques in *Populus*, such as CRISPR/Cas9 (Zhou *et al.*, 2015), single‐nucleotide substitutions and biallelic mutations could be produced to improve our understanding of allelic interactions in poplar.

## Experimental procedures

### Association population and phenotypic data

#### Association population

An association population of 5‐year‐old, 435 unrelated individuals was asexually propagated via root segments in 2011 in Guan Xian County, Shandong Province, China (36°23'N, 115°47'E) in a randomized complete block design with three blocks. The sampling population was randomly selected from a collection of 1,047 natural *P*. *tomentosa* individuals (Du *et al.*, 2014), representing almost the entire natural distribution of *P*. *tomentosa* (30–40°N, 105–125°E). Total genomic DNA was extracted from fresh leaves of each individual using a DNeasy Plant Mini kit (Qiagen, Shanghai, China) following the manufacturer’s protocol.

#### Phenotypic data

To estimate the photosynthetic efficiency and biomass accumulation of plants in the population, 24 photosynthesis‐related traits were measured using functional leaves (i.e., the fourth to sixth leaves from the top of the stem), which were broadly classified into five categories: photosynthetic characteristics, leaf shape, leaf biomass, pigment content and enzyme activities (Table S8). Photosynthetic characteristics were measured using the LI‐6400 portable photosynthesis system (LI‐COR Inc., Lincoln, NE, USA) to measure the net photosynthetic rate (*Photo*), conductance to H_2_O (*Cond*), intercellular CO_2_ concentration (*Ci*) and transpiration rate (*Trmmol*). To reduce the influence of circadian rhythms on photosynthesis, the traits were measured in the functional leaves of all 435 individuals in the association population at 9:00 to 11:00 AM on a sunny day during the growing season in 2016. All measurements were performed using three replications per individual. The leaves that were used to measure photosynthetic characteristics were subsequently used to score leaf shape and biomass. Leaf shape was measured using a CI‐202 portable laser leaf area meter (CID Bio‐Science, Inc., Camas, WA, USA) to characterize leaf length (LL), leaf width (LW), leaf area (LA) and leaf aspect (Aspect). For leaf biomass, after measuring leaf shape traits, the leaves were collected and used to measure fresh weight (LFM) and dried in an oven to constant weight to measure dry weight (LDM). Leaf dry mass per area (LMA) was calculated by the formula: LMA = (leaf dry weight)/(leaf area). To measure pigment content and enzyme activities, functional leaves were collected from 435 individuals. After the photosynthetic characteristics were measured, the leaves were immediately frozen in liquid nitrogen. The pigment contents, including total chlorophyll (Chl), chlorophyll a (Chla), chlorophyll b (Chlb) and carotenoid (Car), were assayed via UV/VIS spectrophotometry according to the manufacturer’s instructions (Komin, Suzhou, China). The wavelength used to deduce the carotenoid (Car) was 440nm, total chlorophyll (Chl), chlorophyll a (Chla), chlorophyll b (Chlb) were 645 nm and 663 nm. To measure enzyme activity, eight enzymes that play important roles in photosynthesis were selected to evaluate the photosynthetic efficiency. These enzymes included fructose‐1,6‐diphosphate (FDP), ferredoxin (FDX), leaf ferredoxin‐NADP^+^ oxidoreductase (LFNR), ribulose bisphosphate carboxylase oxygenase (Rubisco), Rubisco activase (RCA), ribulose‐5‐P‐kinase (Ru5PK), sedoheptulose‐1,7‐diphosphate (SBP) and transketolase (TKT). The methods used to assay enzyme activities were based on an enzyme‐linked immunosorbent assay (ELISA) according to the manufacturer’s instructions (Komin, Suzhou, China).

### RNA isolation, RNA‐seq and lncRNA prediction

Total RNAs were extracted from the mature leaves of 1‐year‐old *P. tomentosa* clones (1316) planted in greenhouse under a 16‐h‐light and 8‐h‐dark cycle (with three independent biological replicates), using the Plant Qiagen RNeasy kit according to the manufacturer’s instructions, which were used for RNA‐seq of mRNAs and lncRNAs. The transcripts’ expression was normalized based on fragments per kilobase of transcript per million fragments (FPKM). The lncRNA prediction was used the pipeline described by Tian *et al. *(2016). The processing of transcriptome data is described in Method S1.

### Coexpression module of photosynthesis‐related PEGs in *Populus*

To identify the PEGs involved in the core photosynthetic pathway, the expressed PEGs in leaves were subjected to GO enrichment analysis with agriGO (Tian *et al.*, 2017) with the false discovery rate (FDR) value cut‐off set to 0.05. The PEGs enriched for GO terms related to the biological process of photosynthesis (GO:0009765, GO:0015979 and GO:0019684) were involved in photosynthesis in poplar species. To search the coexpression module for PEGs in *Populus*, we computed the correlation coefficient based on the expression level of 94 PEGs involving in photosynthesis from all 15 tissues from *Populus*, including the 14 *P. trichocarpa* tissues described by Rodgers‐melnick *et al. *(2012) and one leaf tissue from *P. tomentosa* described above. The Spearman’s rank correlation coefficient (ρ) between the expression patterns of each pair of genes was calculated using Spearman in the R package (Ihaka & Gentleman 1996). A gene pair was considered to exhibit a significant correlation when the estimated positive and negative correlation was |ρ| > 0.95 and *P* value < 0.001, respectively.

### Prediction of the potential miRNAs and lncRNAs for coexpression module

The transcript sequences of photosynthetic PEGs were used as queries to predict the associated miRNAs using the psRNATarget server (Dai & Zhao 2011). In addition, degradome sequencing of pool of six tissues (leaf, shoot apex, phloem, cambium, developing xylem and mature xylem) from *P. tomentosa* was performed to verify the prediction results. The lncRNA data were identified in the RNA‐seq data of ‘1316’ leaves have been reported previously (Tian *et al.*, 2016). The detail methods for predicting miRNAs and lncRNAs for coexpression module genes are described in Method S2.

### Reverse‐transcription quantitative PCR (RT‐qPCR)

Four leaves (the third to sixth leaves from the top of the stem) were collected from 1‐year‐old *P. tomentosa* clone ‘1316’ and immediately frozen in liquid nitrogen. Total RNA was extracted from each leaf and reverse transcribed into cDNA using the Reverse Transcription System (Promega Corporation, Madison, WI, USA). RT‐qPCR was performed on the 7500 Fast Real‐Time PCR System using SYBR Premix Ex Taq (TaKaRa, Dalian, China) according to the manufacturer’s protocol. Primer Express 5.0 software (Applied Biosystems) was used to design specific primers for each gene (for primers used in this study see Table S13). All reactions were performed in three technical and biological repeats with poplar *Actin* (accession number: EF145577) as the internal control and the PCR program described by Xiao *et al. *(2017).

### SNP calling following resequencing of the association population

The 435 individuals of the association population were resequenced at a depth >15× (raw data) using the Illumina GA2 sequencing platform. The Clean reads were mapped to the *Populus* reference genome v3.0, which were used to perform SNP calling (Method S3). The blastall program in BLAST was used to obtain location information for PEGs, lncRNA and miRNA genes (Camacho *et al.*, 2009). VCFtools was used to extract the gene‐derived biallelic SNPs within the full‐length sequence of PEGs and lncRNA, including promoter region (2 kb upstream) and flanking region (500 bp downstream); miRNA genes included pre‐miRNA and the 600 bp flanking sequences on each side of pre‐miRNAs. The detailed methods of SNP calling are described in Method S3.

### SNP‐based associations

The mixed linear model (MLM) in Tassel 5.0 was used to perform single SNP‐trait association analysis between common SNPs (MAF>5%, missing <20%), and the phenotypes were normalized based on the Z‐score. The MLM considers the effects of population structure (*Q*) and kinship coefficients (*K*). The *Q* and *K* matrix were obtained as described by Du *et al. *(2019). QVALUE software in R was used to correct for multiple testing with the positive false discovery rate (FDR) method (Storey, 2003). The significance level of single SNP‐based association results was *P* < 0.001, *q* < 0.05.

### Multi‐SNP epistasis association analysis

The EPISNP1 package in the epiSNP_v4.2 software suite was used to test the epistatic interaction effects of SNP–SNP pairs with phenotypic traits based on the extended Kempthorne model (Ma et al., 2008). This model tests five epistasis effects for each SNP pair, including two‐locus interactions and additive × additive, dominance × dominance, additive × dominance, and dominance × additive effects. The significance level was *P* < 0.001.

### eQTN mapping

eQTN mapping describes the statistically significant correlations of genotype–gene expression levels. RNA‐seq was used to measure the transcript levels of genes from the functional leaves of the 435 unrelated *P. tomentosa* individuals. RNA library construction and sequencing were performed by Beijing Biomarker Technology Cooperation (Beijing, China). The FPKM values (gene expression levels) were calculated as described above. The gene expression traits that were not expressed in> 80% of the 435 individuals were removed prior to eQTL analysis, and log_10 _(1 + FPKM) was used to represent the expression levels of trait‐related genes. eQTL analysis was performed with Tassel 5.0 software using the method described for the SNP‐based association studies. The significance level of the association results was *P* < 0.001, *q* < 0.05.

### Construction of the *PtoPsbX1* overexpression vector and transformation of *A. thaliana*

To grow *A. thaliana* seedlings, seeds were surface‐sterilized and planted on 0.8% agar‐solidified MS medium. After incubation for 4 d at 4°C in darkness, the seeds were transferred to continuous white light at an intensity of 120 mmol/m^2^/s and incubated at 22°C for 7 d. To obtain adult plants, the seedlings were transferred to soil and maintained in a growth chamber with the following conditions: standard long‐day conditions (16 h light/8 h darkness), light intensity of 120 mmol/m^2^/s and temperature of 22 °C.

To construct the *PtoPsbX1* overexpression vector, the coding sequence of *PtoPsbX1* was amplified from *P. tomentosa* clone ‘1316’, cloned into the *pCXSN* vector under the control of the 35S promoter, and introduced into *Agrobacterium* GV3101. The resulting *Agrobacterium* was used to transform wild‐type *A. thaliana* (Col‐0) plants using the floral dip method. Transformants were selected on hygromycin‐containing (50 mmol/L) medium and progressed to the T_2_ generation. The photosynthesis‐related traits and relative expression of *PtoPsbX1* were measured in three independent homozygous transformed lines.

### Accession numbers

The RNA‐seq data for ‘1316’ leaves have been submitted to the NCBI Sequence Read Archive under accession number of SRP060593 and the Genome Sequence Archive in BIG Data Center, Beijing Institute of Genomics (BIG), Chinese Academy of Sciences (CAS) under accession number CRA000992, which is publicly accessible at http://bigd.big.ac.cn/gsa/. The raw data of miRNA sequencing, degradome sequencing, and genome resequencing of 435 *P*. *tomentosa* individuals have been submitted to the Genome Sequence Archive in the BIG Data Center under accession numbers CRA000983, CRA000989 and CRA000903, respectively.

## Author contributions

D.Z. designed the experiments; L.X., X.L., W.L., and M.Q. collected and analysed the data; L.X., X.L., P.C., J.S., and F.S. performed the experiments; L.X. and Q.D. wrote the manuscript; D.Z. obtained funding and is responsible for this article. All authors read and approved the manuscript.

## Supporting information


**Figure S1** The regulatory miRNA target to the PEGs.
**Figure S2** LD decay of candidate genes in the association population of *P. tomentosa*.
**Figure S3** Correlation matrix of 24 photosynthesis‐related traits.
**Figure S4** Manhattan and quantile–quantile plots resulting from the SNP‐based association studies for leaf area (A‐D) and leaf mass (E‐G) traits.
**Figure S5** Manhattan and quantile–quantile plots resulting from the SNP‐based association studies for photosynthetic characterizes (A‐D) and pigment content (E‐H) traits.
**Figure S6** Manhattan and quantile–quantile plots resulting from the SNP‐based association studies for enzyme activity traits (A‐I).
**Figure S7** Interaction network of candidate genes constructed by eQTN mapping.
**Figure S8** Changes in photosynthesis‐related traits in *PtoPsbX1*‐overexpressing *A. thaliana* plants.

**Table S1** GO terms related to photosynthesis for the expressed PEGs in ‘1316’ leaves.
**Table S2** Significant Spearman’s rank correlation coefficients of photosynthesis‐related PEGs.
**Table S3** Annotation information and abbreviated names of candidate PEGs.
**Table S4** LncRNA–PEG pairs identified in this study.
**Table S5** MiRNA–PEG pairs identified in this study.
**Table S6** Detailed information of the single‐nucleotide polymorphisms (SNPs) within all candidate genes.
**Table S7** Detailed information of the nucleotide polymorphisms within all candidate genes.
**Table S8** Phenotypic variation of 24 photosynthesis‐related traits in the association population of *P. tomentosa*.
**Table S9** Phenotypic correlations for 24 photosynthesis‐related traits in the association population of *P. tomentosa*.
**Table S10** Detailed information of the significant SNPs within candidate genes associated with photosynthesis‐related traits in the association population of *P. tomentosa*.
**Table S11** Detailed information of the significant epistatic SNP–SNP pairs for each trait in the association population of *P. tomentosa*.
**Table S12** Detailed information of the eQTNs identified for each gene in the photosynthetic coexpression network.
**Table S13** Gene‐specific primers used in this study.

**Method S1** RNA isolation, RNA‐seq, long noncoding RNAs (lncRNAs) prediction.
**Method S2** Prediction of the potential miRNAs and lncRNAs for coexpression module.
**Method S3** SNP calling following resequencing of the association population.
