# Supplementary material for: Genetic dissection of the gene coexpression network underlying photosynthesis in Populus
Source: Plant Biotechnol J. 2019 Oct 21;18(4):1015–26. doi: 10.1111/pbi.13270 (PMC7061883; doi:10.1111/pbi.13270)
Supplement: Supplementary file 1 — Figure S1 The regulatory miRNA target to the PEGs. Figure S2 LD decay of candidate genes in the association population of P. tomentosa. Figure S3 Correlation matrix of 24 photosynthesis‐related traits. Figure S4 Manhattan and quantile–quantile plots resulting from the SNP‐based association studies for leaf area (A‐D) and leaf mass (E‐G) traits. Figure S5 Manhattan and quantile–quantile plots resulting from the SNP‐based association studies for photosynthetic characterizes (A‐D) and pigment content (E‐H) traits. Figure S6 Manhattan and quantile–quantile plots resulting from the SNP‐based association studies for enzyme activity traits (A‐I). Figure S7 Interaction network of candidate genes constructed by eQTN mapping. Figure S8 Changes in photosynthesis‐related traits in PtoPsbX1‐overexpressing A. thaliana plants. [file PBI-18-1015-s002.doc]

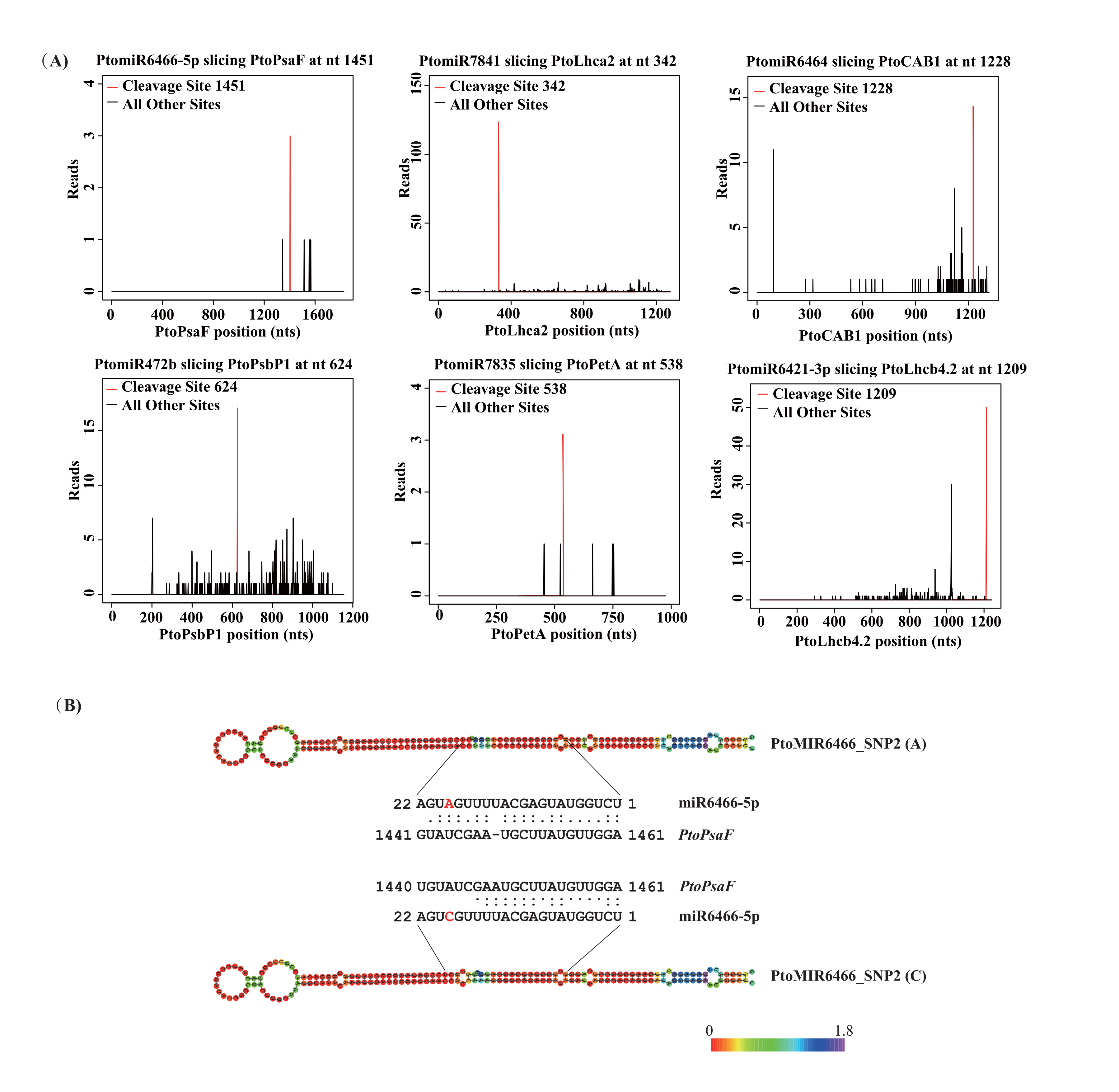


**Figure S1 The regulatory miRNA target to the PEGs** (A) The miRNA cleavage sites in their corresponding target genes identified by degradome sequencing. The red vertical bars indicate the most likely cleavage sites. (B) PtoMIR6466_SNP2 in the mature region of *PtomiR6466* destabilized its secondary structure which were increased a loop and altered the minimum free energy, which increased from  −81.80 to  −77.10 kcal/mol. PtoMIR6466_SNP2 also altered the mature product of *PtomiR6466*, preventing it from interacting with its target gene (*PtoPsaF*). The dots between the sequences indicate complementary base pairing in the binding site. PtoMIR6466_SNP2(A) and PtoMIR6466_SNP2(C) are alleles of PtoMIR6466_SNP2.


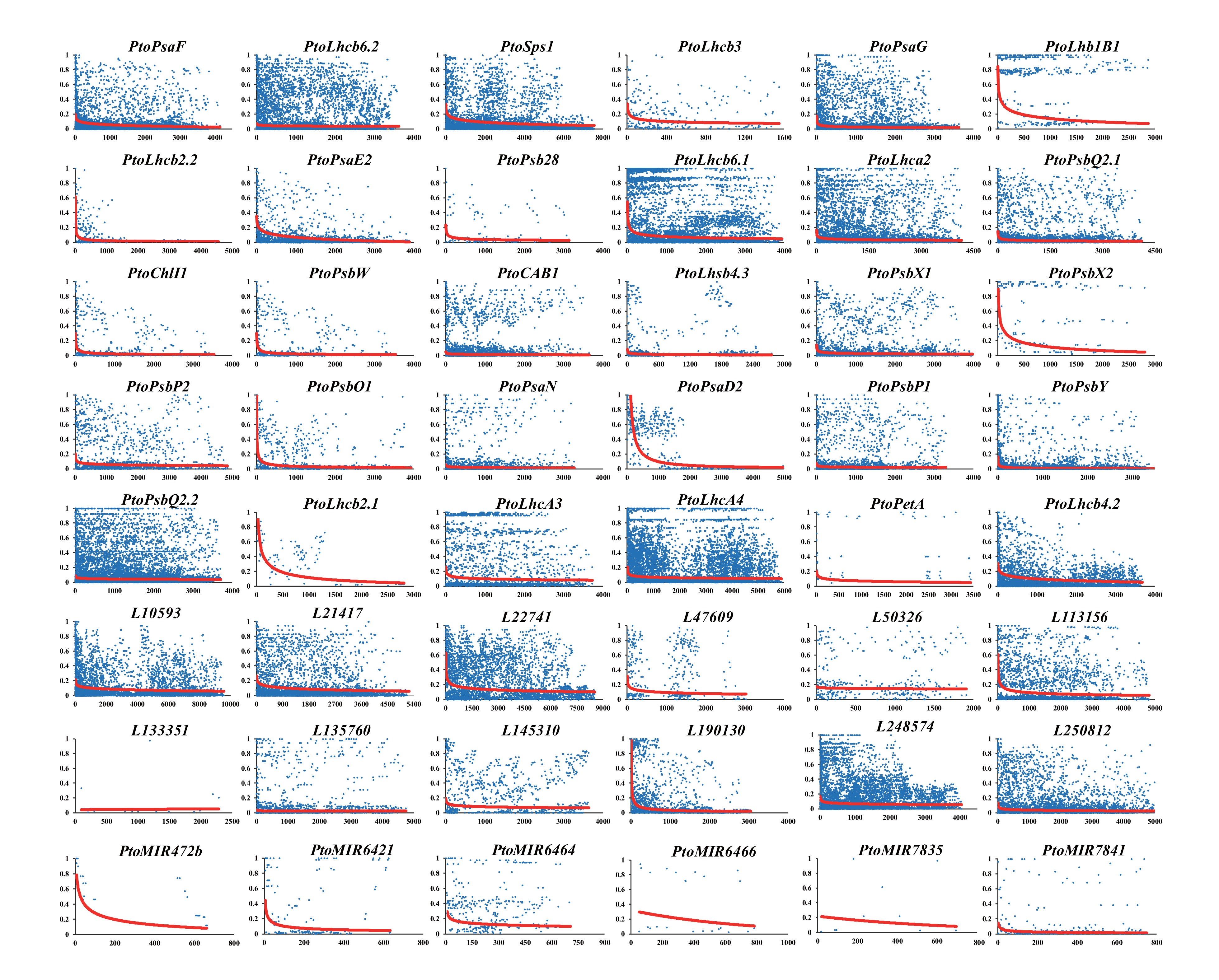


**Figure S2 LD decay of candidate genes in the association population of *P. tomentosa*.** Nonlinear regressions of *r*^2^ versus physical distances are described by separate curves for each gene. The x- and y-axes represented the physical distances and *r*^2^, respectively.


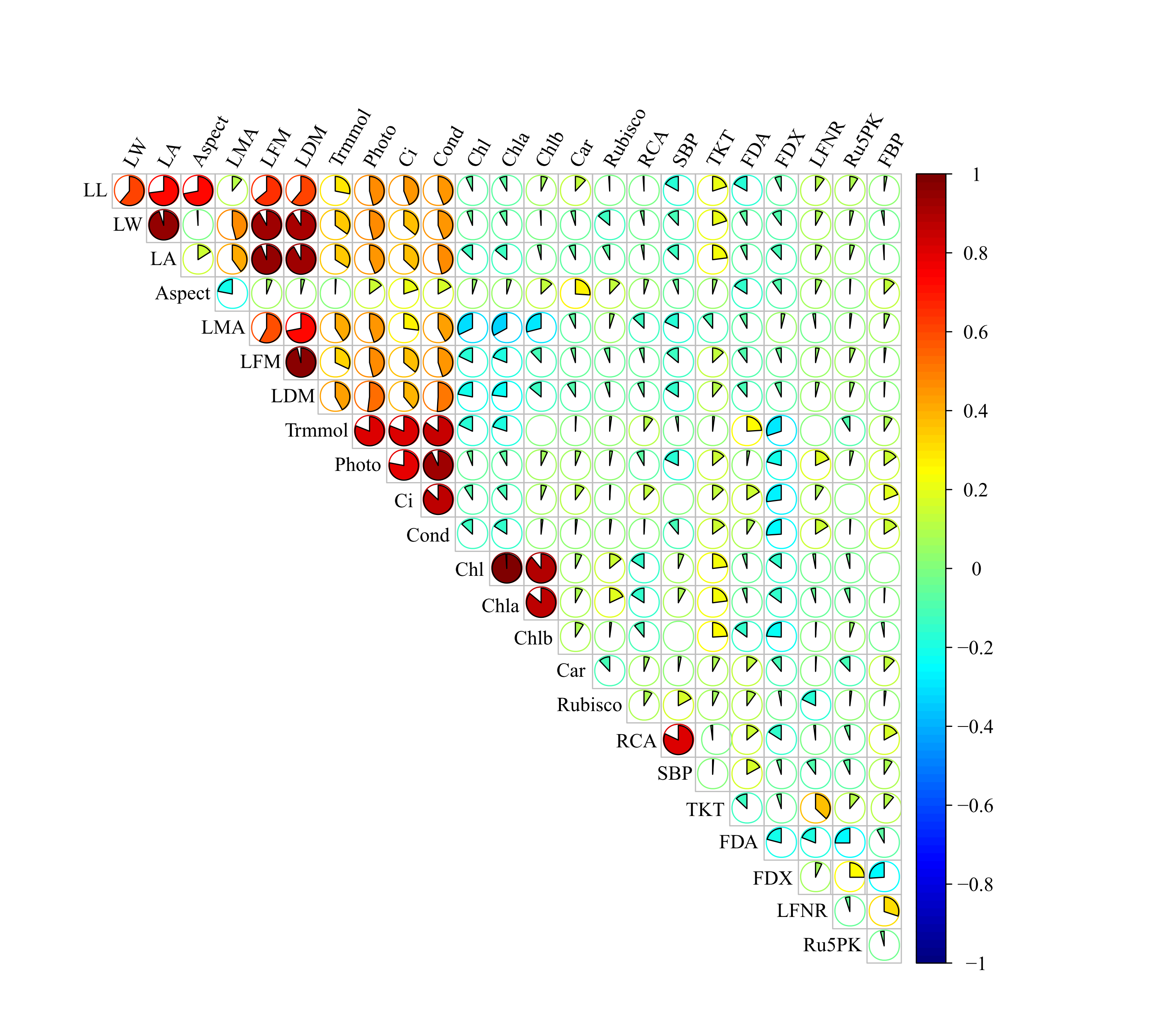


**Figure S3 Correlation matrix of 24 photosynthesis-related traits.** Blue indicates negative correlations and red indicates positive correlations. The filled portion of each pie chart is proportional to the strength of the correlation.


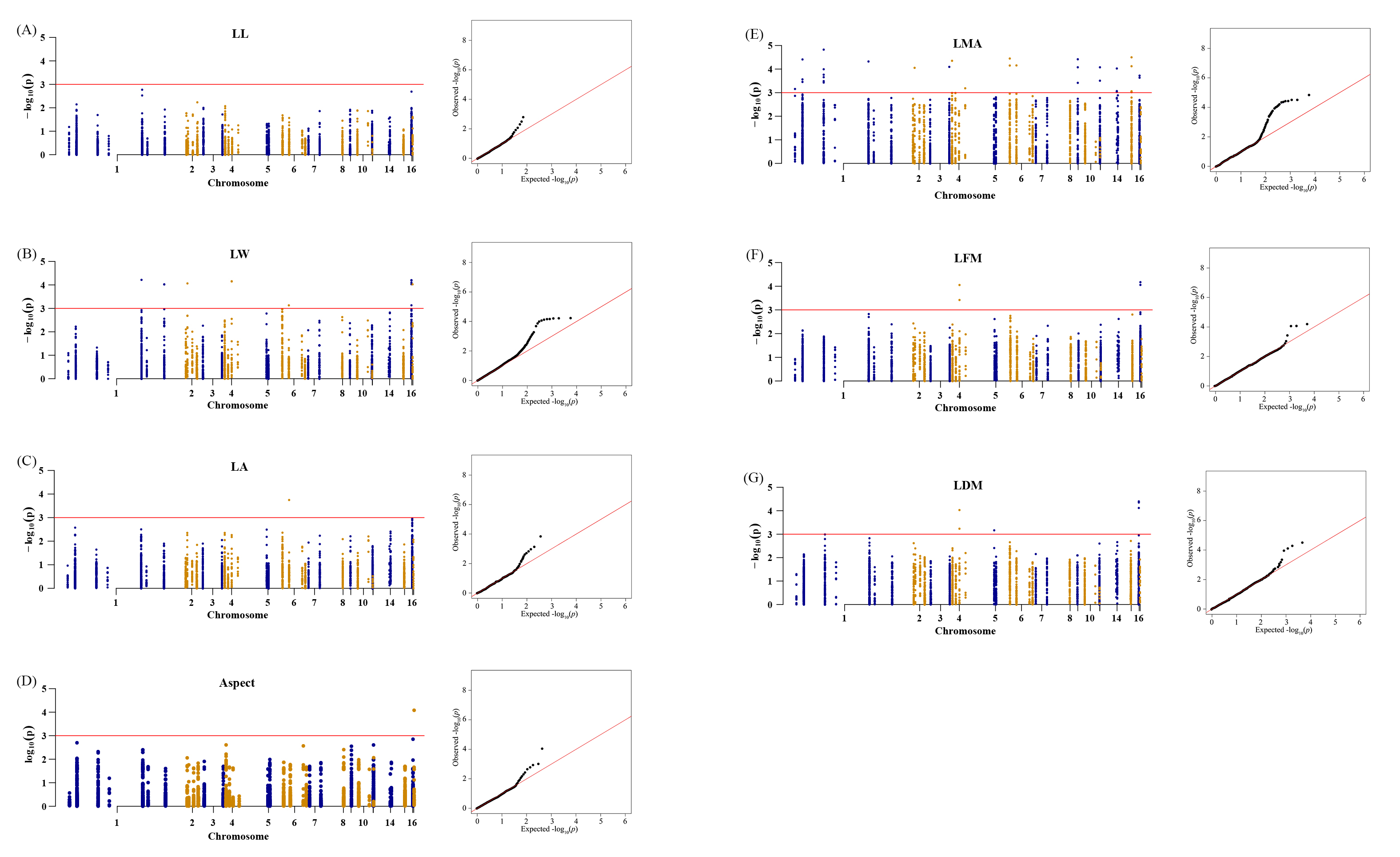


**Figure S4 Manhattan and quantile-quantile plots resulting from the SNP-based association studies for leaf area (A-D) and leaf mass (E-G) traits.** The red line in each Manhattan plot represented the significance threshold (*P* = 0.001). The *x* and *y* axis showed the genomic position and the Negative log_10_ *P*-values.


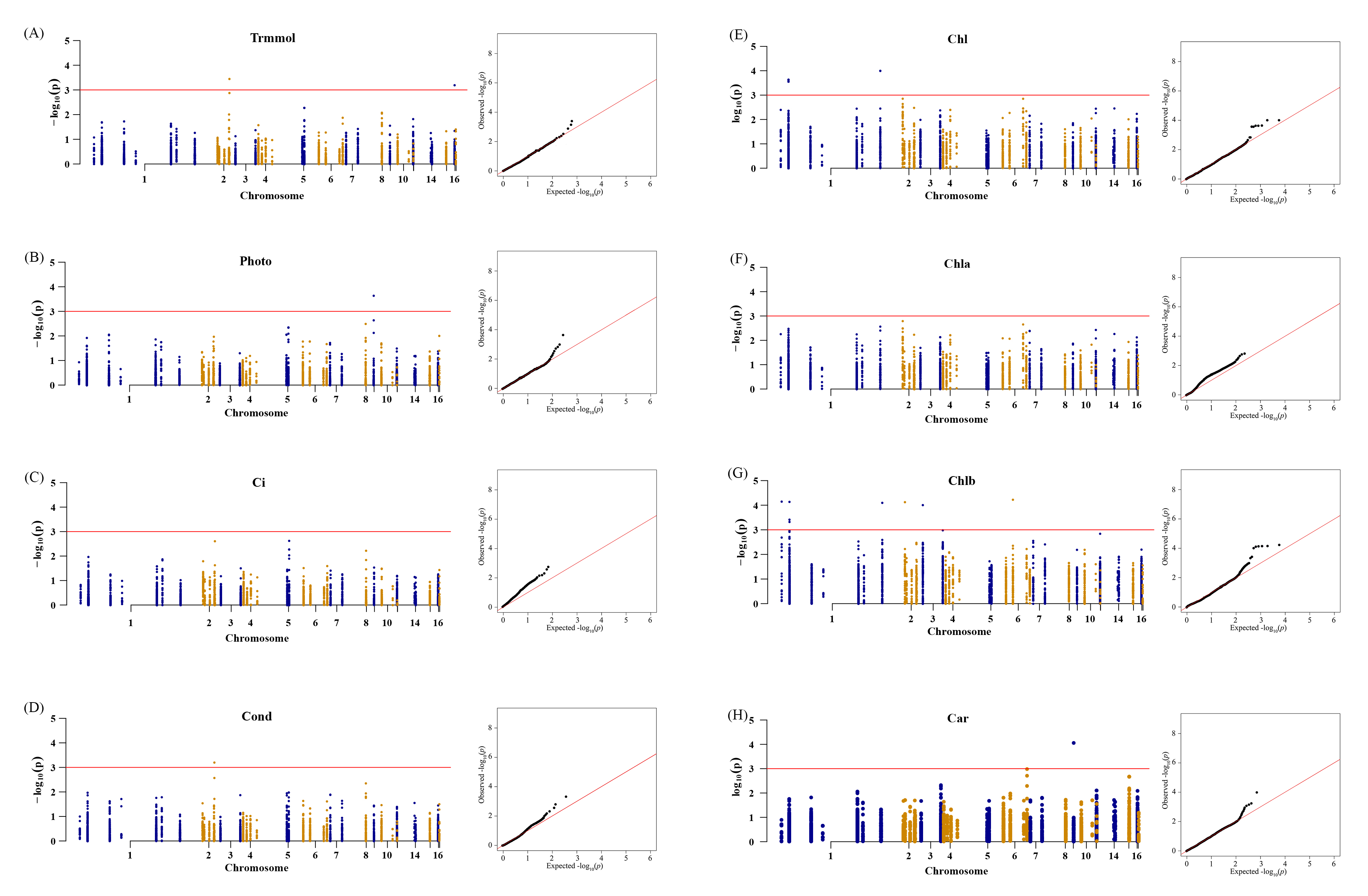


**Figure S5 Manhattan and quantile-quantile plots resulting from the SNP-based association studies for photosynthetic characterizes (A-D) and** **pigment content (E-H) traits.** The red line in each Manhattan plot represented the significance threshold (*P* = 0.001). The *x* and *y* axis showed the genomic position and the Negative log_10_ *P*-values.


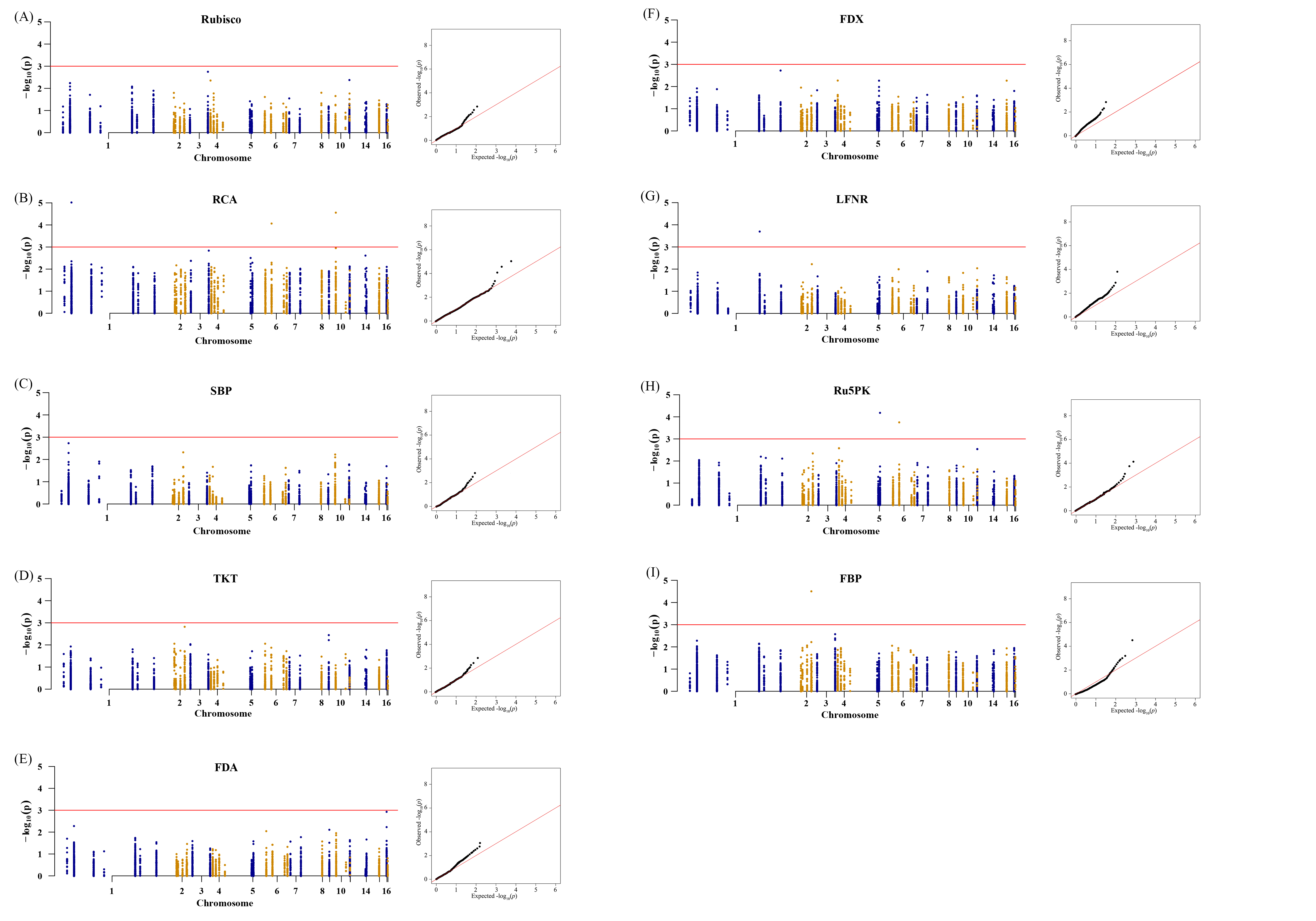


**Figure S6 Manhattan and quantile-quantile plots resulting from the SNP-based association studies for enzyme activity traits (A-I).** The red line in each Manhattan plot represented the significance threshold (*P* = 0.001). The *x* and *y* axis showed the genomic position and the Negative log_10_ *P*-values.


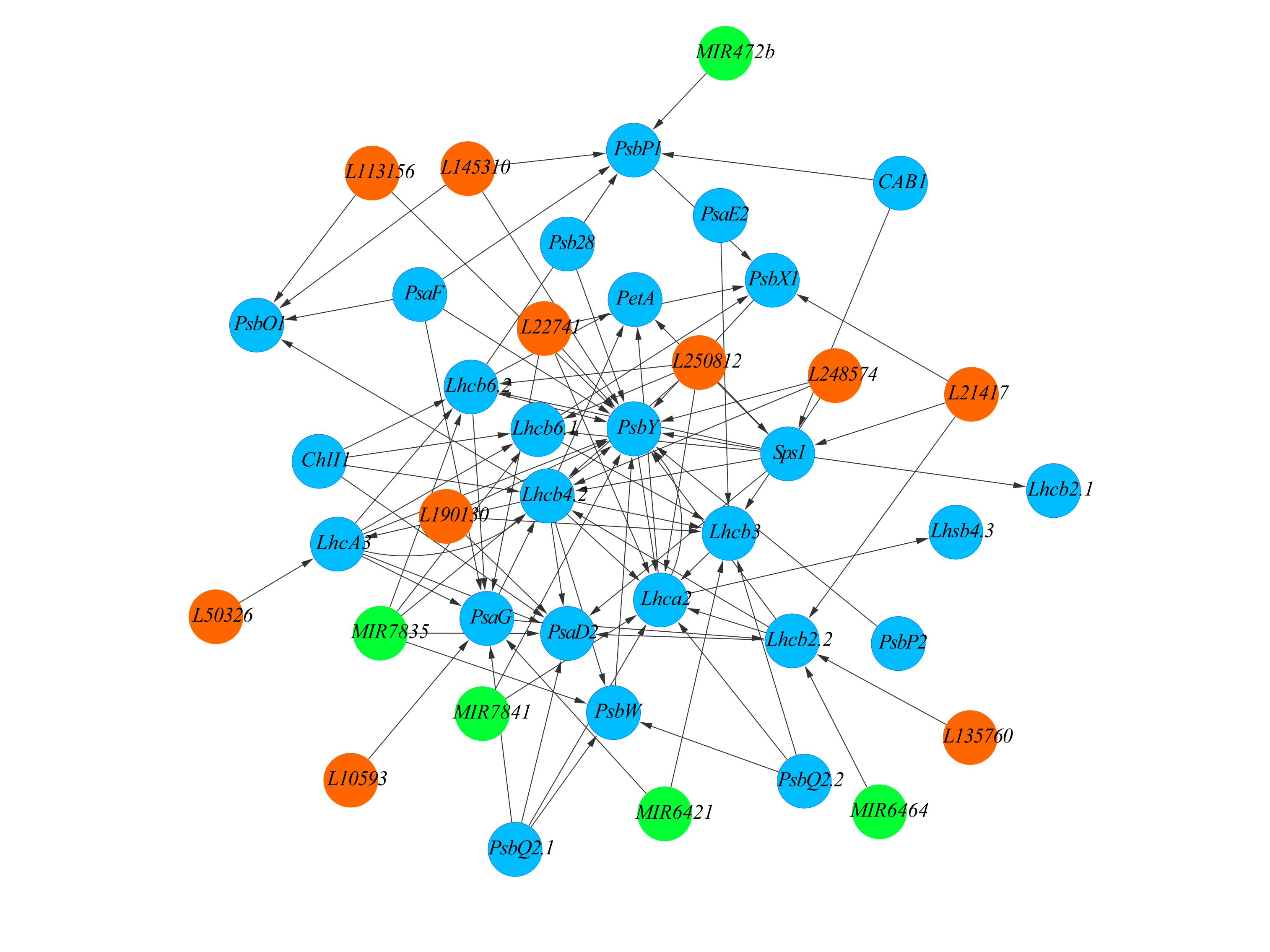


**Figure S7 Interaction network of candidate genes constructed by eQTN mapping.** The blue, orange, and green circles represent the PEG, lncRNA, and miRNA genes, respectively. The arrows pointing from gene 1 to gene 2 indicate that the SNPs in gene 1 regulate the expression of gene 2.


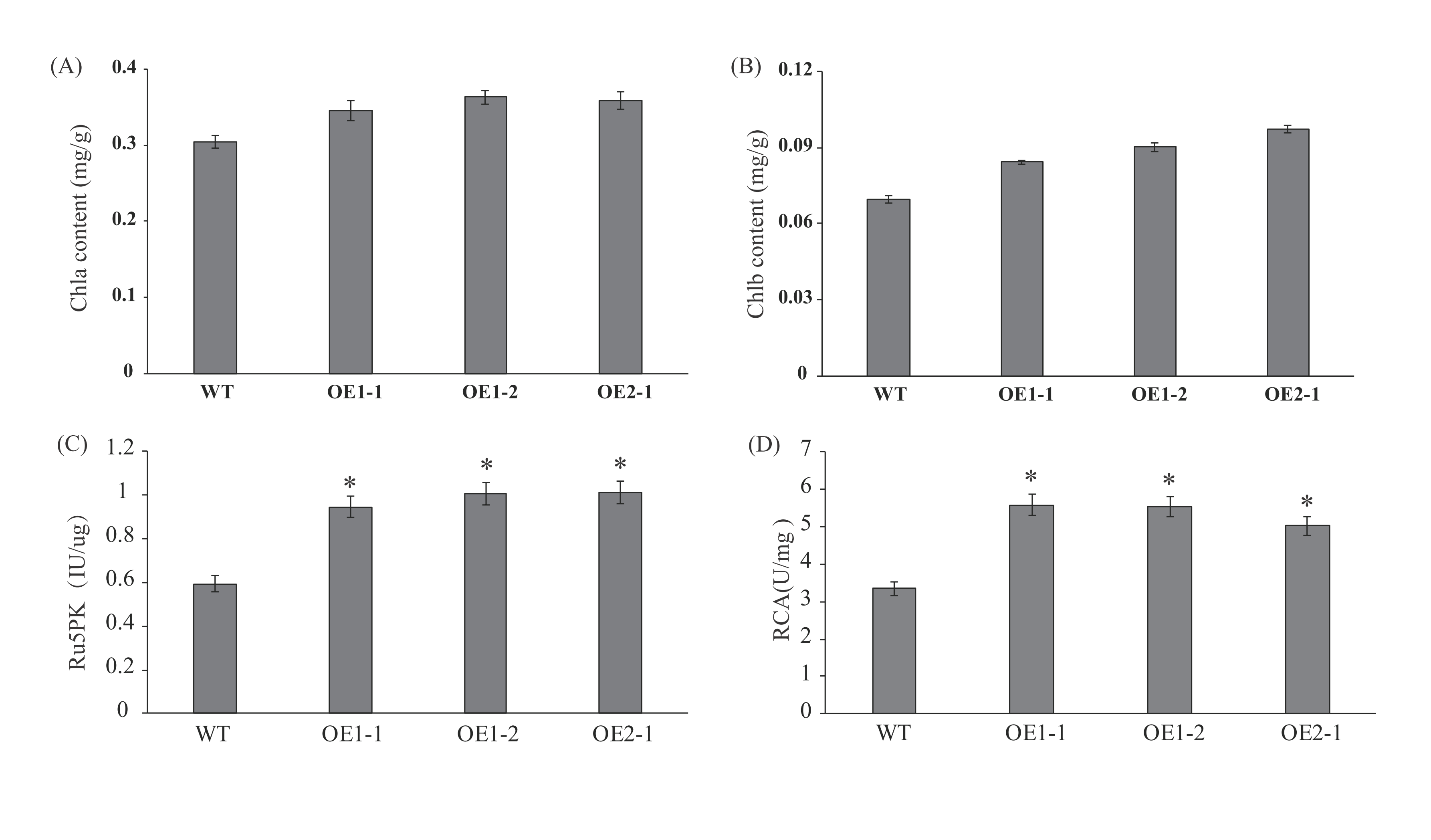


**Figure S8 Changes in photosynthesis-related traits in *PtoPsbX1*-overexpressing *A. thaliana* plants.** The chlorophyll a (Chla) content (A), chlorophyll b (Chlb) content (B), Ru5PK activity (C), and RCA activity (D) in the wild type (WT) and three overexpression lines. *Significant differences from the WT based on Student’s *t*-test (*P* < 0.01); n = 15.
