# Supplementary material for: Genetic dissection of the gene coexpression network underlying photosynthesis in Populus
Source: Plant Biotechnol J. 2019 Oct 21;18(4):1015–26. doi: 10.1111/pbi.13270 (PMC7061883; doi:10.1111/pbi.13270)
Supplement: Supplementary file 3 — Method S1 RNA isolation, RNA‐seq, long noncoding RNAs (lncRNAs) prediction. Method S2 Prediction of the potential miRNAs and lncRNAs for coexpression module. Method S3 SNP calling following resequencing of the association population. [file PBI-18-1015-s003.doc]

**Method S1 RNA isolation, RNA-seq, long nocoding RNAs (lncRNAs) prediction**

Mature leave (with three independent biological replicates) were collected from 1-year-old *P. tomentosa* clones (1316) grown in a greenhouse under a 16-h-light and 8-h-dark cycle and immediately frozen in liquid nitrogen. Total RNA was extracted from the samples with a Qiagen RNeasy kit according to the manufacturer’s instructions (Qiagen China, Shanghai, China). In addition, DNase digestion was performed at the time of RNA purification using an RNase Free DNase set (Qiagen). The RNA sample were assessed with a NanoDrop ND-1000 (A260/A280 = 1.91) and Agilent Bioanalyzer 2100 (28S/18S = 1.6). A total of 3 μg RNA per sample was used to build construct strand-specific RNA-seq library using the NEBNext® UltraTM RNA Library Prep Kit for Illumina® (NEB, USA) following manufacturer’s recommendations and index codes. The clustering of the index-coded samples was performed on a cBot Cluster Generation System using TruSeq PE Cluster Kit v3-cBot-HS (Illumia) according to the manufacturer’s instructions. After being quantified with a Qubit 2.0 Fluorometer and Agilent 2100 Bioanalyzer, the strand-specific libraries were sequenced on an Illumina HiSeq 2500 instrument, which generated 100-nt paired-end reads. Library construction and paired-end sequencing were performed by Shanghai Biotechnology Corporation (Shanghai, China). Raw data were downloaded from the NCBI Sequence Read Archive (accession number SRP060593, CRA000992). To obtain the clean data, the reads containing adapter, reads containing ploy-N and low quality reads were removed from raw data by in-house perl scripts.

The reference genome (*P. trichocarpa* genome Version 3.0) and genomic annotations were downloaded from Phytozome (<http://www.phytozome.net/>). Next, the clean data were uniquely mapped to the reference genome using TopHat 2.1.1 with the default options (Trapnell et al., 2009). The isoform levels and gene level counts of the assembled transcripts were computed and normalized based on fragments per kilobase of transcript per million fragments (FPKM) values using Cufflinks v2.1.1 with default options (Trapnell et al., 2012).

Then, the clean data were also used to predict lncRNAs using the pipeline described by Tian et al. (2016). In brief, transcripts with mapping coverage of less than half the transcript length, FPKM < 1, shorter than 200 bp, or encoded by open reading frames longer than 100 amino acids were discarded. The coding potentials of the remaining transcripts were evaluated using Coding Noncoding Index (CNCI) software (http://www.bioinfo.org/software/cnci) (Sun et al., 2013) and coding potential calculator (CPC) software (http://cpc.cbi.pku.edu.cn/) (Kong et al., 2007), using the protein-coding transcripts of *P. trichocarpa* as a reference. All transcripts with CNCI > 0 or CPC scores > 0 were discarded. The lncRNAs were classified into intergenic, intronic, antisense, and sense lncRNAs using the Cuffcompare program in the Cufflinks suite.

**Method S2 Prediction of the potential miRNAs and lncRNAs for coexpression module**

The transcript sequences of photosynthetic PEGs were used as queries to predict the associated miRNAs using the psRNATarget server (Dai et al., 2011). In addition, degradome sequencing was performed to verify the psRNATarget results. Six tissues (leaf, shoot apex, phloem, cambium, developing xylem, and mature xylem) were collected from *P. tomentosa*, and total RNA was extracted and pooled together in equal amounts. The pooled RNA sample were used to build the degradome library for degradome sequencing on the Illumina HiSeq2000 platform; the detailed methods were described by Zhou et al. (2010). Finally, based on the *P. trichocarpa* genome transcripts (v 3.0) (SRX1447192), the miRNA cleavage sites were identified according to the CleaveLand pipeline (Addo-Quaye et al., 2009).

To predict the potential target genes of the lncRNAs, two independent algorithms were used based on the regulatory effects of the lncRNAs (*cis*- and *trans*-acting). For *cis-*acting lncRNAs, the potential target genes were identified based on the physical distance between each lncRNA and mRNA gene. If the distance was <10 kb, the gene was considered to be a potential *cis* target gene of the corresponding lncRNA (Jia et al., 2010). The potential *trans* targets were predicted using an algorithm based on sequence complementarity and RNA duplex energy predictions; the detailed criteria have been described by Tian et al. (2016).

## Method S3 SNP calling following resequencing of the association population

The 435 individuals of the association population were resequenced at a depth >15× (raw data) using the Illumina GA2 sequencing platform according to the manufacturer’s instructions. Raw reads were trimmed through a series of quality control (QC) procedures. QC standards as the following: (1) Removing reads with ≥10% unidentified nucleotides (N); (2) Removing reads with > 50% bases having phred quality < 5; (3) Removing reads with > 10 nt aligned to the adapter, allowing ≤ 10% mismatches; (4) Removing putative PCR duplicates generated by PCR amplification in the library construction process (read 1 and read 2 of two paired-end reads that were completely identical).

Then, the clean reads of each sample were aligned to the *Populus* reference genome using SOAPaligner (SOAP2, version 2.20) with default parameters (Li et al., 2009). The mapping rates of individuals ranged from 81% to 92%, and the effective mapping depth was ~15× for most individuals. The uniquely mapped pair-end reads were used for SNP calling. Polymorphisms at each potential SNP site were carefully examined using the following criteria: (1) At least four unique reads and must support the base pair call; (2) Each SNP must have at least a quality value of 20 (< 1% error rate); (3) SNPs must have two and only two alleles; (4) The alternative allele must be supported by at least 80% of all aligned reads covering that position.

To verify the accuracy of the results of SNP calling, the data were compared with our previous SNP data from 10 candidate genes from 120 individuals discovered by PCR-Sanger sequencing. The accuracy of SNP calling was 97.5%, indicating that the SNP-calling platform was of high quality. Following imputation, SNPs with a minor allele frequency < 0.05 and a missing data > 0.2 were removed by VCFtools (Danecek et al., 2009). The blastall program in BLAST was used to obtain location information for the photosynthetic PEGs, lncRNA genes, and miRNA genes (Camacho et al., 2009). VCFtools was used to extract the gene-derived biallelic SNPs within the full-length, photosynthetic, and lncRNA genes, including their 2-kb upstream and 500-bp downstream sequences. The miRNA genes included 1-kb flanking sequences on each side of the pre-miRNA as the primary transcript sequences. In addition, the snpEff software was used to annotate the SNPs to different genomic annotation categories, which including synonymous and non-synonymous in exon region (Cingolani et al., 2012).

**References:**

Addo-Quaye C, Miller W, Axtell M J (2008) CleaveLand: a pipeline for using degradome data to find cleaved small RNA targets. *Bioinformatics* **25**: 130-131.

Camacho C, Coulouris G, Avagyan V, et al (2009) BLAST+: architecture and applications. *BMC bioinformatics* **10**: 421.

Cingolani P, Platts A, Wang L L, et al (2012) A program for annotating and predicting the effects of single nucleotide polymorphisms, SnpEff: SNPs in the genome of Drosophila melanogaster strain w1118; iso-2; iso-3. Fly 6: 80-92.

Danecek P, Auton A, Abecasis G, et al (2011) The variant call format and VCFtools. *Bioinformatics* **27**: 2156-2158.

Dai X, Zhao P X (2011) psRNATarget: a plant small RNA target analysis server. *Nucleic Acids Research* **39**: W155-W159.

Jia H, Osak M, Bogu G K, et al (2010) Genome-wide computational identification and manual annotation of human long noncoding RNA genes. *RNA* **16**: 1478-1487.

Kong L, Zhang Y, Ye Z Q, et al (2007) CPC: assess the protein-coding potential of transcripts using sequence features and support vector machine. *Nucleic Acids Research* **35**:W345-W349.

Li R, Yu C, Li Y, et al (2009) SOAP2: an improved ultrafast tool for short read alignment. *Bioinformatics* **25**: 1966-1967.

Sun L, Luo H, Bu D, et al (2013) Utilizing sequence intrinsic composition to classify protein-coding and long non-coding transcripts. *Nucleic Acids Research* **41**: e166-e166.

Tian J, Song Y, Du Q, et al (2016) Population genomic analysis of gibberellin-responsive long non-coding RNAs in *Populus*. *Journal of Experimental Botany* **67**: 2467-2482.

Trapnell C, Roberts A, Goff L, et al (2012) Differential gene and transcript expression analysis of RNA-seq experiments with TopHat and Cufflinks. *Nature Protocols* **7**: 562-578.

Trapnell C, Pachter L, Salzberg S L (2009) TopHat: discovering splice junctions with RNA-Seq. *Bioinformatics* **25**: 1105-1111.

Zhou M, Gu L, Li P, et al (2010) Degradome sequencing reveals endogenous small RNA targets in rice (*Oryza sativa* L. ssp. indica). *Frontiers in Biology* **5**: 67-90.
